# Extracellular vesicles derived from hypoxic glioma stem-like cells confer temozolomide resistance on glioblastoma by delivering miR-30b-3p

**DOI:** 10.7150/thno.47057

**Published:** 2021-01-01

**Authors:** Jianxing Yin, Xin Ge, Zhumei Shi, Chen Yu, Chenfei Lu, Yutian Wei, Ailiang Zeng, Xiefeng Wang, Wei Yan, Junxia Zhang, Yongping You

**Affiliations:** 1 Department of Neurosurgery, The First Affiliated Hospital of Nanjing Medical University, Nanjing, China.; 2 Institute for Brain Tumors, Jiangsu Key Lab of Cancer Biomarkers, Prevention and Treatment, Jiangsu Collaborative Innovation Center for Cancer Personalized Medicine, Nanjing Medical University, Nanjing, China.; 3 Department of Nutrition and Food Hygiene, Center for Global Health, School of Public Health, Nanjing Medical University, Nanjing, China.; 4 Department of Cancer Biology, The University of Texas M.D. Anderson Cancer Center, Houston, Texas 77030, USA.

**Keywords:** GSCs, extracellular vesicles, miR-30b-3p, hypoxia, TMZ, chemoresistance

## Abstract

**Rationale:** Glioma stem-like cells (GSCs) contribute to temozolomide (TMZ) resistance in gliomas, although the mechanisms have not been delineated.

**Methods:** *In vitro* functional experiments (colony formation assay, flow cytometric analysis, TUNEL assay) were used to assess the ability of extracellular vesicles (EVs) from hypoxic GSCs to promote TMZ resistance in glioblastoma (GBM) cells. RNA sequencing and quantitative Reverse Transcription-PCR were employed to identify the functional miRNA in hypoxic EVs. Chromatin immunoprecipitation assays were performed to analyze the transcriptional regulation of miRNAs by HIF1α and STAT3. RIP and RNA pull-down assays were used to validate the hnRNPA2B1-mediated packaging of miRNA into EVs. The function of EV miR-30b-3p from hypoxic GSCs was verified by *in vivo* experiments and analysis of clinical samples.

**Results:** Hypoxic GSC-derived EVs exerted a greater effect on GBM chemoresistance than those from normoxic GSCs. The miRNA profiling revealed that miR-30b-3p was significantly upregulated in the EVs from hypoxic GSCs. Further, HIF1α and STAT3 transcriptionally induced miR-30b-3p expression. RNA immunoprecipitation and RNA-pull down assays revealed that binding of miR-30b-3p with hnRNPA2B1 facilitated its transfer into EVs. EV-packaged miR-30b-3p (EV-miR-30b-3p) directly targeted RHOB, resulting in decreased apoptosis and increased proliferation *in vitro* and *in vivo*. Our results provided evidence that miR-30b-3p in CSF could be a potential biomarker predicting resistance to TMZ.

**Conclusion:** Our findings indicated that targeting EV-miR-30b-3p could provide a potential treatment strategy for GBM.

## Introduction

Glioblastoma (GBM) is the most common primary malignant brain tumor in adults 1. Despite the current treatment of maximal surgical resection, radiotherapy, and chemotherapy, the prognosis for newly diagnosed GBM patients remains poor with a median survival of less than two years 2, 3. Temozolomide (TMZ) is used as first-line chemotherapy for GBM treatment. However, glioma stem-like cells (GSCs), a subset of cancer cells capable of self-renewal 4-6, limit GBMs' effective treatment due to their therapeutic resistance 7, 8. The generation and maintenance of GSCs require a supportive niche. The surrounding stromal cells, such as reactive astrocytes, immune cells, and endothelial cells, are considered components of the GSC 9, 10 niche.

Hypoxia has been postulated to induce resistance to radio- or chemotherapeutic treatment in several malignant tumors 11, 12. The stabilization and activation of hypoxia-inducible factor (HIF) proteins, especially HIF1α and HIF2α, are considered important cellular responses to hypoxia. HIF1α and HIF2α function as gene activators for malignant tumor generation and evolution 13, 14. GSCs often reside in hypoxic microenvironments, and it is hypothesized that hypoxia supports the maintenance of their undifferentiated state and therapeutic resistance 15, 16. However, the underlying mechanisms of hypoxia maintenance of GSC stemness are unknown.

Extracellular vesicles (EVs) shed by tumor cells serve as important mediators of intercellular communication, thus influencing carcinogenesis 17. EV-mediated communication between tumor cells has gained increasing awareness in the field, with many studies focusing on the impact of tumor or stromal cell-derived EVs 18, 19. EVs also proved to be involved in the microenvironment reprogramming 20, 21. However, the role of EVs released from GSCs is poorly understood. Herein, we sought to investigate EV-mediated communication of GSCs in different microenvironments. We hypothesized that GSCs maintained in a hypoxic environment release EVs containing specific elements that may confer therapeutic resistance to GBM.

## Materials and Methods

### Clinical samples

Sixty GBM samples were collected from patients who underwent surgical resection in The Department of Neurosurgery, The First Affiliated Hospital of Nanjing Medical University. The clinical characteristics of patients are detailed in Supplementary Table 1. This study was approved by the Institutional Review Board and the Ethics Committee of Nanjing Medical University (Ethics number: 2019-SR-479), and written informed consent was obtained from all patients.

### Cell culture

We obtained five paired primary tumor cells (TCs) and GSCs, and each paired TCs and GSCs were obtained from the same GBM sample. GBM surgical specimens were dissociated into single cells using Accutase® (Sigma-Aldrich, St. Louis, MO, USA) to obtain TCs, which were maintained in Dulbecco^'^s Modified Eagle's Medium (DMEM) supplemented with 10% fetal bovine serum (FBS). To obtain GSCs, cells from fresh GBM surgical specimens were cultured in neurobasal media (Invitrogen) supplemented with B27 (Invitrogen, Carlsbad, CA, USA), epidermal growth factor, and fibroblast growth factor-2 (Invitrogen). GSCs were sorted with a magnetic sorting system with CD133 as the surface marker (Figure S1A). All cells were maintained at 37 °C in 5% CO_2_ and replenished every three months from frozen stocks. The O6-methylguanine-DNA methyltransferase (MGMT) promoter methylation patterns were analyzed as shown in Table S2.

Immunofluorescence and Western blot assays confirmed the expression of GSC markers CD133 and Nestin (Figure S1B-C). The expression of stem cell markers, Sox2 and Olig2, and the absence of GFAP in GSCs were assessed by Western blotting quantitative real-time PCR (qRT-PCR) analysis (Figure S1C and D). *In vitro* limiting dilution assays were performed to assess the sphere-forming ability of GSCs 22. Briefly, different numbers of pared GSCs were seeded into 96-well ultra-low attachment microplates (Corning, USA) and maintained under neurobasal conditions for 7 days, and the percent of wells without spheres were plotted against the number of cells in each well (Figure S1E). Furthermore, we transfected matched TCs and GSCs with constitutive luciferase reporter to monitor the tumor volume. The results showed that GSC injection led to faster tumor growth and poor survival rate (Supplementary Figure 1F-H). We also identified the expression of GFAP (astrocyte marker) and CD31 (endothelial cell marker) in tumors derived from TCs and GSCs (Supplementary Figure 1I). Together, these data provided further evidence for the GSC identification.

The TMZ IC50 was much higher in GSCs than the corresponding GBM cells (Figure S1J), suggesting that GSC664 and GSC712 are more resistant to TMZ than TC664 and TC712. To simulate the tumor hypoxic environment, cells were cultured under 1% O_2_, balanced with N_2_ in a three-gas incubator (Binder).

### Conditioned medium (CM) preparation

1×10^6^ GSC664 and GSC712 cells were cultured under normoxia or hypoxia for 48h, the culture medium was collected and supplemented with 2% exosome-free FBS (SBI, USA). 1×10^6^ TCs were cultured using this medium.

### Isolation of EVs

Normoxic or hypoxic GSC-derived EVs were isolated by the differential centrifugation of conditioned GSC medium 18. Briefly, GSC664 and GSC712 cells were maintained in a normoxic or hypoxic environment for 48 h. The conditioned medium was then collected and differentially centrifuged at 300 g for 10 min, 1,000 × g for 20 min, and 10,000 × g for 30 min at 4 °C. Next, the supernatant was filtered using a 0.22 µm filter unit (Millex-GP; EMD Millipore, Darmstadt, Germany) and ultra-centrifuged at 100,000 × g for 3 h at 4 °C. After removal of the supernatant, the pellets were washed in ice-cold PBS. The suspension was centrifuged at 100,000 × g for another 3 h at 4 °C. EV pellets were either resuspended in PBS for further experiments or stored at -80 °C. BCA (bicinchoninic acid assay, KenGEN, Jiangsu, China) was used to assess the concentration of EVs. EVs were visualized by scanning electron microscopy (SEM) and confirmed by the expression of EV-specific proteins CD63 and CD81 17.

### RNA isolation and qRT-PCR

Total, cytoplasmic, and nuclear RNA were extracted using TRIzol reagent according to the manufacturer's instructions 23, 24. EV-RNA extraction was performed, as previously described 24. A stem-loop-specific primer method was used to measure miR-30b-p expression, as described previously 25. The cDNA was amplified by qRT-PCR using SYBR® Premix Ex Taq™ (Takara, Kusatsu, Japan) on a 7900HT system (Applied Biosystems/Thermo Fisher Scientific, Foster City, CA, USA). Fold-changes were calculated by relative quantification (2-ΔΔCt). The primers used for PCR are presented in Supplementary Table 3.

### Protein extraction and Western blot analysis

Protein extraction and Western blot analysis were performed as previously reported 19, 23. Briefly, cells or glioma tissues were lysed in the lysis buffer supplemented with protease inhibitors for 30 min on ice. The lysates were centrifuged at 14,000 × g at 4 °C for 15 min, the supernatants were collected, and protein concentrations were determined using the BCA methods. Protein samples were separated by SDS-PAGE and transferred onto PVDF membranes, which were blocked with 5% non-fat dried milk for 2 h and incubated with primary antibodies overnight at 4 °C. An electrochemiluminescence detection system (Thermo Fisher Scientific) was used for detection. All immunoblots were performed at least three times. The antibodies used for Western blot analysis are listed in Table S4.

### Drugs

Dimethyl sulfoxide (DMSO) was used to dissolve TMZ powder (Sigma-Aldrich) to a final concentration of 100 mM.

### Colony formation assay

TC664 and TC712 after different treatments were seeded in six-well plates at a density of 400 cells/well. Cells were treated with 200 μM TMZ every three days. Two weeks later, cell colonies were fixed and stained with crystal violet. We defined a colony as 50 cells.

### Flow cytometric analysis

Cell apoptosis was analyzed using the annexin V-fluorescein isothiocyanate Apoptosis Detection Kit I (BD Biosciences), and the apoptotic rate was evaluated by fluorescence-activated cell sorting (FACS).

### Terminal deoxynucleotidyl transferase dUTP nick end labeling (TUNEL) analysis

TUNEL analysis was performed using the detection kit (Millipore) according to the manufacturer's instructions.

### miRNA library construction and sequencing

Total RNAs were isolated from EVs, which were obtained from GSC664 and GSC712 cultured in normoxia or hypoxia for 48h. RNAs ranging from 18 to 30 nucleotides (nt) were employed for constructing the miRNA library. miRNAs were reverse transcribed and amplified using PCR and sequenced by the Illumina HiSeq 2500 system.

### RNA Immunoprecipitation (RIP) Assay

Briefly, cells were resuspended in radio-immunoprecipitation assay (RIPA) buffer and incubated with 20 μL protein beads and 1 μg antibody complex overnight at 4 °C. After incubation with RNase-free DNase I (Promega) at 37 °C for 15 min and proteinase K at 45 °C for 45 min, RNA samples were extracted with 1 mL Trizol and subjected to RT-PCR analysis.

### Chromatin Immunoprecipitation (ChIP) Assay

ChIP assay was performed using the Simple Chip Enzymatic Chromatin IP kit (Cell Signaling Technology, Danvers, MA, USA) following the manufacturer's instructions. Briefly, cell chromatin was incubated with 3 µg anti-HIF1α or STAT3. Purified immunoprecipitated DNA was prepared for the PCR with specific primers (Table S3).

### Biotin-labeled pull-down assays

Biotinylated miR-30b-3p pull-down assays were performed with target proteins. Briefly, 1 × 10^6^ cells were prepared a day before transfection. Next day, 3' biotin-labeled wild-type or mutant miR-30b-3p was transfected at a final concentration of 50 nM. Plasma lysates were harvested after 48 h. Streptavidin-Dyna beads (Dyna beads M-280 Streptavidin, #11205D, Invitrogen, 50 μL each sample) were coated with 10 μL per sample yeast tRNA (stock 10 mg/ml Ambion, Austin, TX, USA) and incubated with rotation at 4 °C for 2 h. The beads were then washed and resuspended in the lysis buffer. Plasma lysates were mixed with pre-coated beads and incubated on a rotator at 4 °C overnight. After washing, the retrieved protein was subjected to Western blot analysis.

### Animal study

Male athymic nude mice (nu/nu) were purchased from the Vital River Animal Center (Beijing, China) and maintained in special pathogen-free (SPF) conditions. Intracranial xenografts were implanted as previously described 18. To assess chemoresistance, mice were intraperitoneally injected with 20 mg/kg TMZ following a standard schedule of 4 weeks on and 4 weeks off treatment from the second week post-surgery. All animal manipulations were performed in accordance with the ethics guidelines of Nanjing Medical University.

### Statistical analysis

All data were analyzed using GraphPad Prism 6. The two-tailed Student's t-test was employed to assess the significance of differences. The survival analysis was performed using log-rank tests. *P* < 0.05 was considered statistically significant.

## Results

### EVs released from hypoxic GSCs promote TMZ resistance in GBM cells

Hypoxic conditioned media (HCM) and normoxic conditioned media (NCM) from GSC664 and GSC712 cells were used to culture their corresponding TCs. *In vitro* functional experiments showed that HCM significantly increased TMZ resistance in GBM cells compared to NCM (Figure S2 A-D). EVs were eliminated via a sequential filtration (SF) protocol, as previously described 26. As shown in Figure S2A-D, EVs depletion significantly reduced the pro-chemoresistance effect of HCM, suggesting that EVs from hypoxic GSCs may play an important role in acquired chemoresistance of GBM cells.

**Figure 1:**
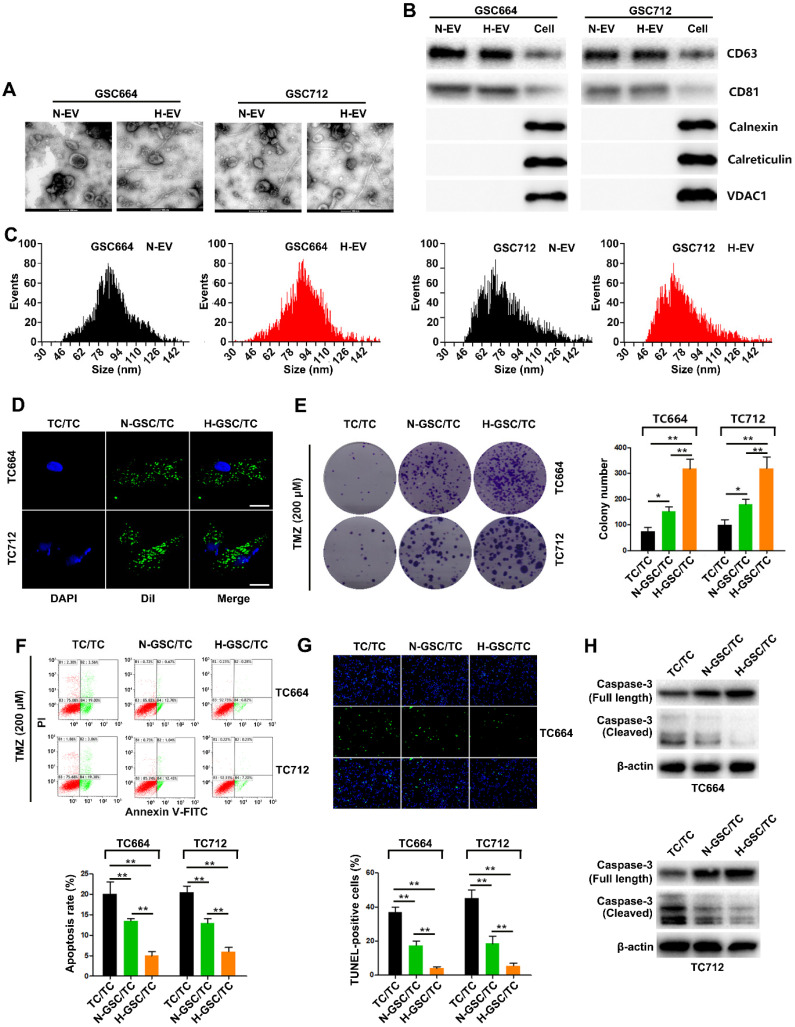
** EVs released from hypoxic GSCs promote chemoresistance in GBM cells.** (A) Representative images of normoxic EVs (N-EV) and hypoxic EVs (H-EV). Scale bar, 100 nm. (B) Western blot analysis of markers of EVs in N-EV, H-EV and cells. (C) NTA results of N-EV and H-EV of GSCs. (D) Representative confocal microscopy images of EV internalization by GBM cells. Scale bar, 10 µm. (E) Colony formation assays of TC664 and TC712 cells treated with EVs (25 µg/mL) from TC664 or TC712 cells, normoxic GSCs, or hypoxic GSCs (derived EVs/recipient cells). Six replicates per group, three independent experiments per group. (F) Apoptotic rates of TC664 and TC712 cells treated with EVs (25 µg/mL) from TC664 or TC712 cells, normoxic GSCs, or hypoxic GSCs were measured by flow cytometry (derived EVs/recipient cells). Six replicates per group, three independent experiments per group. (G) Apoptotic rates of TC664 and TC712 cells treated with EVs (25 µg/mL) from TC664 or TC712 cells, normoxic GSCs, or hypoxic GSCs were measured by TUNEL analysis (derived EVs/recipient cells). Six replicates per group, three independent experiments per group. (H) Western blot analysis of caspase-3 (full length and cleaved) in TC664 and TC712 cells treated with EVs (25 µg/mL) from TC664 and TC712 cells, normoxic GSCs, or hypoxic GSCs (derived EVs/recipient cells). Three replicates per group, three independent experiments per group, **P < 0.01.

SEM analysis of GSC-derived EVs showed that both normoxic (N-EV) and hypoxic (H-EV) EVs were typical rounded particles (Figure 1A). We confirmed the presence of EVs by Western blot analysis of CD63 and CD81 (Figure 1B). The purified EVs were labeled with fluorescent Dil, and their internalization by GBM cells 2 h after incubation was monitored by fluorescence microscopy (Figure 1C). As shown in Figure 1D-G, *in vitro* functional data showed a significant increase of TMZ resistance in TC664 and TC712 cells after coculturing with EVs from hypoxic GSCs. Furthermore, without TMZ treatment, CM of hypoxic GSCs could also significantly promote the growth of TCs. And as expect, H-EV could promote the growth of TCs (Figure S2E-F) Together, these results suggested that EVs derived from hypoxic GSCs promote TMZ chemoresistance of GBM cells.

### MiR-30b-3p is overexpressed in hypoxic EVs of GSCs

**Figure 2:**
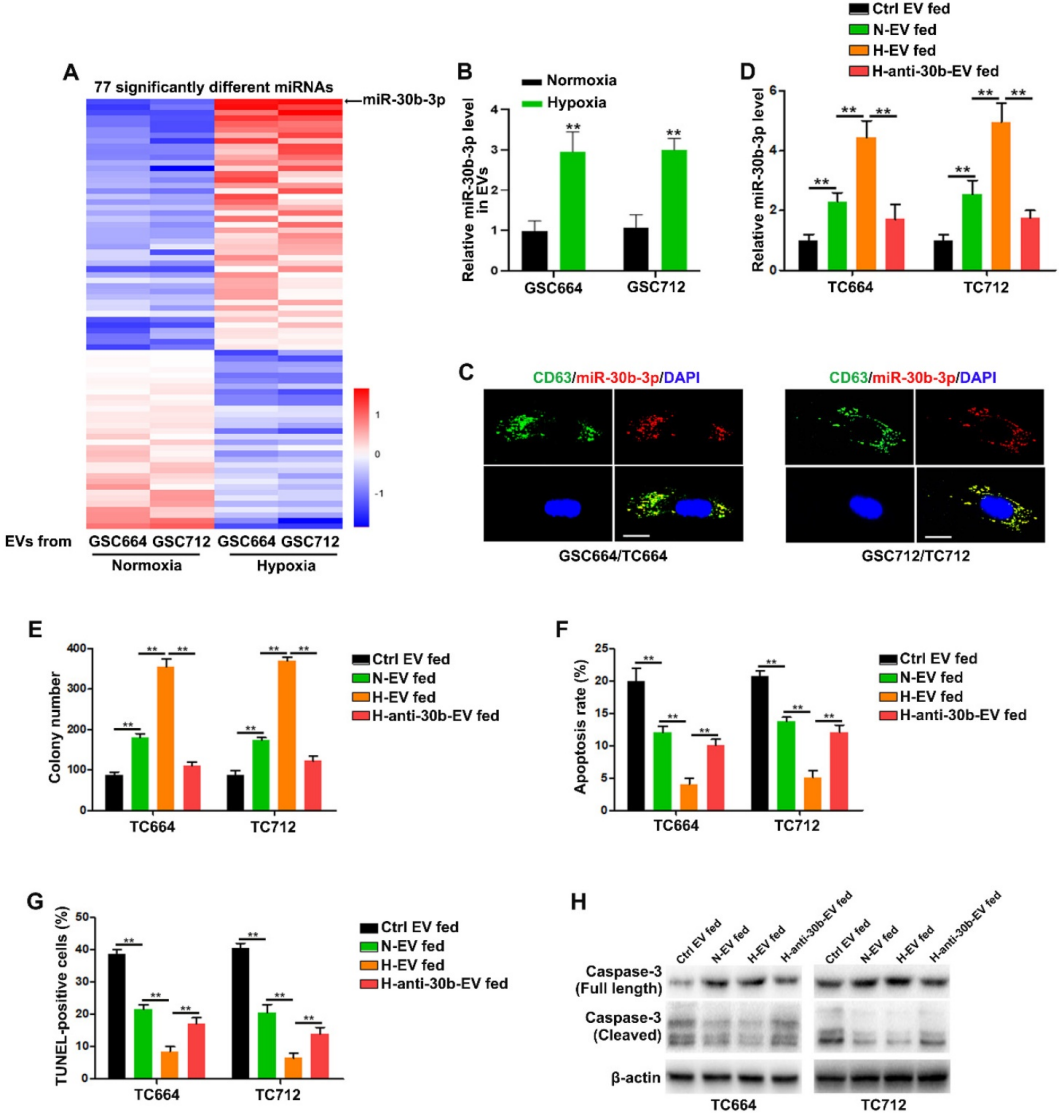
** MiR-30b-3p is functionally overexpressed in hypoxic EVs.** (A) 77 miRNAs were significantly different between normoxic EVs and hypoxic EVs. (B) qRT-PCR analysis of miR-30b-3p in EVs from GSC664 and GSC712 cells under normoxia or hypoxia. Six replicates per sample, three independent experiments per sample. (C) Representative confocal microscopy images of EV-miR-30b-3p internalization by TC664 and TC712 cells. Scale bar, 10 µm. (D) qRT-PCR analysis of miR-30b-3p in TC664 and TC712 cells treated with different EVs. Six replicates per sample, three independent experiments per sample. (E) Colony formation assay of TC664 and TC712 cells treated with different EVs. Six replicates per group, three independent experiments per group. (F) Apoptotic rates of TC664 and TC712 cells treated with different EVs were measured by flow cytometry. Six replicates per group, three independent experiments per group. (G) Apoptotic rates of TC664 and TC712 cells treated with different EVs were measured by TUNEL analysis. Six replicates per group, three independent experiments per group. (H) Western blot analysis of caspase-3 (full length and cleaved) in TC664 and TC712 cells treated with different EVs. Three replicates per group, three independent experiments per group, **P < 0.01.

It has been shown that miRNAs, as important elements of EVs, can be transferred to recipient cells via EVs 24, 27. We employed Illumina HiSeq 2500 high-throughput sequencing for miRNA expression profiling of EVs from normoxic and hypoxic GSCs to identify the functional components in hypoxic EVs. With a 2-fold change and P < 0.05 as the threshold cutoff, we identified 77 significantly differentially expressed miRNAs, of which 45 were upregulated in hypoxic EVs (Figure 2A). Among them, miR-30b-3p was the most significantly increased miRNA in hypoxic EVs compared to normoxic EVs. Consistent with miRNA-seq, qRT-PCR showed that EVs derived from hypoxic GSCs had significantly increased miR-30b-3p levels compared with those derived from normoxic GSCs (Figure 2B and Figure S3B). As expected, GSCs expressed a higher level of miR-30b-3p than TCs (Figure S3A).

To determine whether miR-30b-3p could be transferred from GSCs to GBM cells via EVs, we co-transfected GSC664 and GSC712 cells with lentiviral RFP-CD63 and Cy5-miR-30b-3p. Co-localization of Cy5 and RFP in GBM cells was observed following incubation with EVs secreted by transfected GSC664 and GSC712 cells (Figure 2C). Meanwhile, the miR-30b-3p levels in TC664 and TC712 cells were significantly elevated after incubation with normoxic and hypoxic EVs (Figure 2D). To further confirm the transfer of miR-30b-3p by EVs, we transfected GSC664 and GSC712 with anti-miR-NC or anti-miR-30b-3p. The results showed that the expression of miR-30b-3p was down-regulated in both cells and EVs in either normoxic and hypoxic environments (Figure S3B-C). The expression of miR-30b-3p in recipient GBM cells significantly reduced with a decrease in EVs (Figure 2D), confirming the entrance of EV-miR-30b-3p into GBM cells. Furthermore, *in vitro* experiments showed that down-regulation of miR-30b-3p in hypoxic EVs could abolish the oncogenic function of EVs from hypoxic GSCs (Figure 2 E-H). Collectively, the data suggest that EV-miR-30b-3p is a critical component in GSC EVs remolded by hypoxia.

### HIF1α/pSTAT3 co-activator complex induces expression of miR-30b-3p in hypoxic GSCs

**Figure 3:**
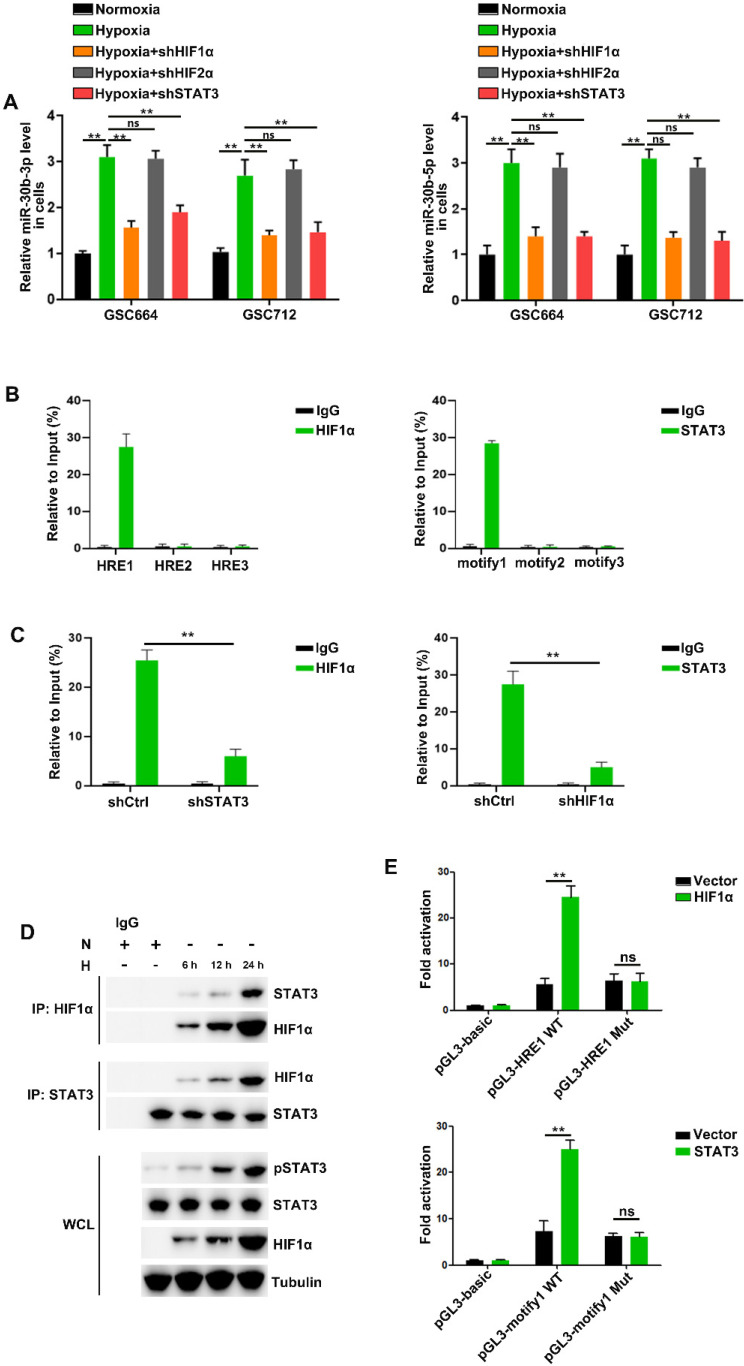
** The HIF1α/pSTAT3 co-activator complex induces miR-30b-3p expression in hypoxic GSCs.** (A) qRT-PCR analysis of miR-30b-3p (left) and miR-30b-5p (right) in GSC664 and GSC712 cells treated with shctrl or shHIF1α or shSTAT3. Six replicates per group, three independent experiments per group. (B) qRT-PCR analyses of immuno-precipitates using primers flanking the HRE sites (left) or STAT3 binding motif (right). Six replicates per group, three independent experiments per group. (C) qRT-PCR analyses of immuno-precipitates using primers flanking the HRE sites after STAT3 knockdown (left) or STAT3 binding motif after HIF1α knockdown (right). Six replicates per group, three independent experiments per group. (D) Co-IP results of HIF1α and STAT3 in GSCs. (E) Luciferase reporter assay was used to detect the MIR30B promoter reporter activity in GSC664 and GSC712 cells transfected with vector or HIF1α or STAT3. Six replicates per group, three independent experiments per group, **P < 0.01.

Consistent with previous studies 16, 28-30, we found that HIF1α, HIF2α and STAT3 were induced by hypoxia in GSC664 and GSC712 cells (Figure S4A) and cellular miR-30b-3p and miR-30b-5p were significantly upregulated (Figure 3A). Further, the hypoxia induction of miR-30b-3p and miR-30b-5p was abolished following HIF1α or STAb3 but not HIF2α downregulation (Figure 3A and Figure S4B), and the expression of pri-miR-30b and pre-miR-30b echoed (Figure S4C). The results suggested potential transcriptional regulation of MIR30B by HIF1α and STAT3.

Analysis of the MIR30B promoter region using the JASPAR database identified three putative HIF1α and three pSTAT3 binding sites on its promoter region (Figure S4D). The CHIP assay indicated that HIF1α bound to MIR30B promoter at -1386 to -1379 bp region and pSTAT3 bound to the miR-30b promoter at -1828 to -1818 bp region (Figure 3B and Figure S4E). HIF1α and STAT3 were previously described to function as a complex to induce gene transcription 31, 32. In this study, we found that hypoxia induced the interaction between HIF1α and STAT3 in TCs (Figure 3D). STAT3 knockdown reduced HIF1α binding to the MIR30B promoter, and conversely, HIF1α knockdown reduced STAT3 binding to the MIR30B promoter (Figure 3C). Further, we cloned wild-type and mutant fragments from the MIR30B promoter into the upstream region of a luciferase reporter (pGL3-basic) (Figure S4F) and found that pGL3-HRE1 WT but not pGL3-HRE1 Mut was activated by HIF1α overexpression. Similar results were obtained in STAT3 overexpressed cells (Figure 3E and Figure S4G). Together, these results suggested that HIF1α and STAT3 complex is required for miR-30b-3p transcription in GSCs under hypoxic conditions.

### HnRNPA2B1 mediates the packaging of miR-30b-3p into EVs

**Figure 4:**
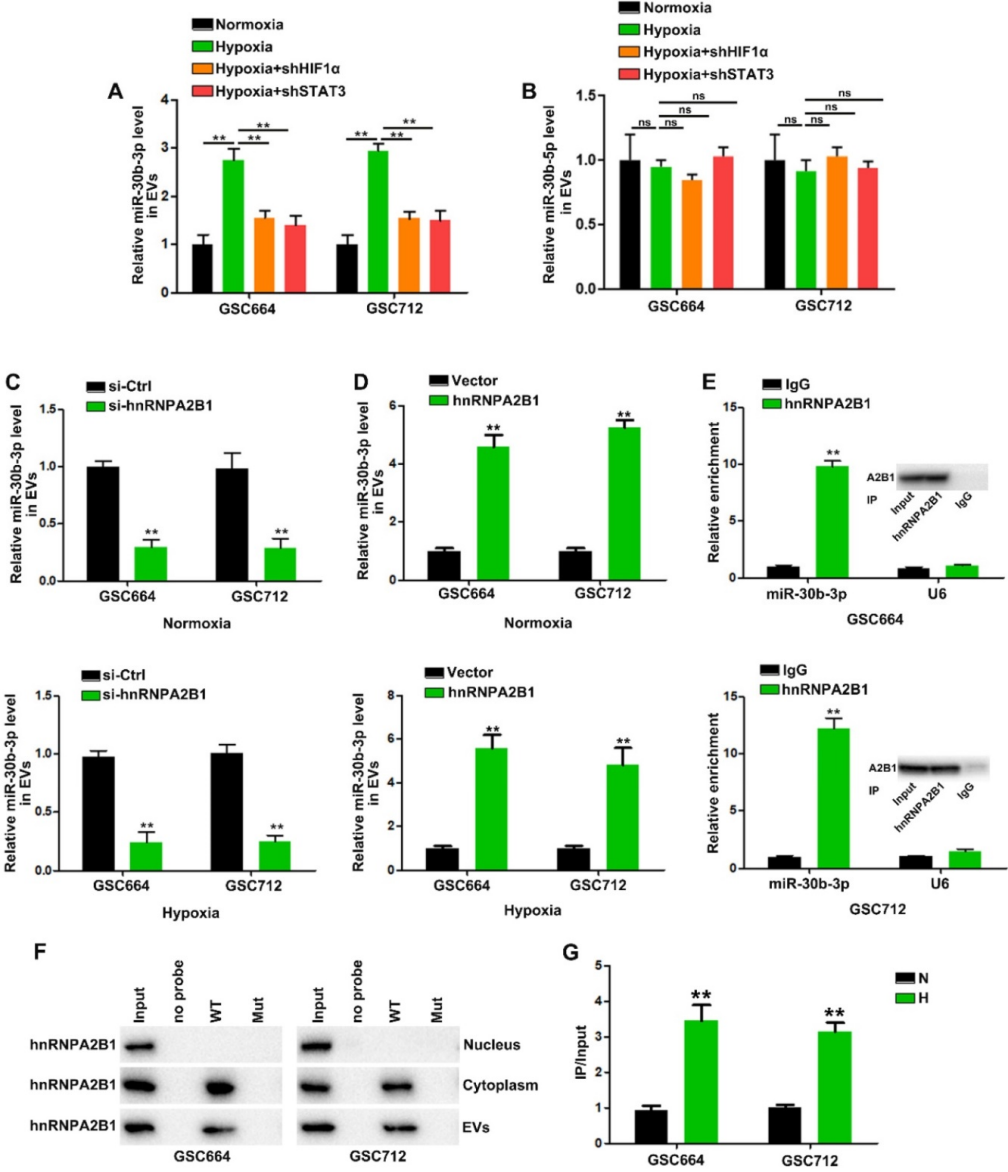
** HnRNPA2B1 mediates the packaging of miR-30b-3p into EVs.** (A) qRT-PCR analysis of miR-30b-3p in EVs from GSC664 and GSC712 cells treated with shHIF1α or shSTAT3. Six replicates per group, three independent experiments per group. (B) qRT-PCR analysis of miR-30b-5p in EVs from GSC664 and GSC712 cells treated with shHIF1α or shSTAT3. Six replicates per group, three independent experiments per group. (C) qRT-PCR analysis of miR-30b-3p in normoxic and hypoxic EVs from GSC664 and GSC712 cells transfected with si-Ctrl or si-hnRNPA2B1. Six replicates per group, three independent experiments per group. (D) qRT-PCR analysis of miR-30b-3p in normoxic and hypoxic EVs from GSC664 and GSC712 cells transfected with vector or hnRNPA2B1. Six replicates per group, three independent experiments per group. (E) GSC664 and GSC712 cells were subjected to RIP assay using an anti-hnRNPA2B1 antibody or IgG. qRT-PCR was used to analyze miR-30b-3p enrichment in IP-enriched RNA. Six replicates per group, three independent experiments per group. (F) Immunoblotting for hnRNPA2B1 in samples pulled down with WT miR-30b-3p, Mut miR-30b-3p or no probe. Three replicates per group, three independent experiments per group. (G) Relative miR-30b-3p levels in IP products of hnRNPA2B1 from normoxic or hypoxic GSCs. Three replicates per group, three independent experiments per group, **P < 0.01.

Next, we investigated the molecular mechanism by which EV-miR-30b-3p was packaged in GSCs. In view of the above results, we analyzed whether hypoxia could induce pri-/pre-miR-30b in EVs. However, little pri-/pre-miR-30b was found in EVs (data not shown). So far, it is not clear whether the incorporation of miRNAs into EVs occurs at the pre-miRNA or mature miRNA level. Our results suggested that only mature miRNA could be packaged into EVs. Figure 4A shows that HIF1α and STAT3 knockdown abolished hypoxia's effect on EV-miR-30b-3p. However, down-regulation of HIF1α and STAT3 did not affect miR-30b-5p levels in EVs (Figure 4B). Since miR-30b-3p and 5p were both induced by HIF1a and STAT3, we speculated that elevated miR-30b-3p may be more efficiently packaged into EVs than miR-30b-5p.

HnRNPA2B1 was reported to be a critical mediator regulating miRNA trafficking to EVs 33, 34. We detected hnRNPA2B1 expression in N-EV and H-EV (Figure S5F). Using miRBase, we found that miR-30b-3p contained a specific motif (GGAG), considered to be one of the two probable hnRNPA2B1 binding sites. Then, we localized miR-30b-3p, miR-30b-5p in GSCs by immunofluorescence and hnRNPA2B1 by Western blotting. MiR-30b-3p and miR-30b-5p were primarily localized in the cytoplasm (Figure S5A-B) and hnRNPA2B1 was present in the nucleus and cytoplasm (Figure S5D-E).

EV-miR-30b-3p levels were significantly decreased after silencing hnRNPA2B1 in either normoxic or hypoxic GSCs (Figure 4C), but neither hnRNPA2B1 knockdown nor its overexpression could affect miR-30b-5p entry into EVs (Figure S5G-H). The qRT-PCR analysis revealed that the hnRNPA2B1 antibody pulled down significantly more miR-30b-3p than the IgG control from the cytoplasmic extracts (Figure 4E). Subsequently, we carried out the RIP of GSC664 and GSC712 cytoplasmic and nuclear extracts and did not detect miR-30b-5p in the IP product of hnRNPA2B1 (data not shown). We then sought to determine whether the miR-30b-3p transcript region containing GGAG mediated interaction with hnRNPA2B1. In miRNA pull-down assays, miR-30Bb-3p showed its specific interaction with hnRNPA2B1 (Figure S5C). We also observed an interaction between miR-30b-3p and hnRNPA2B1 EVs; however, mutation of the GGAG sequence (Figure S5M) significantly impaired the binding capacity of hnRNPA2B1 (Figure 4F). In addition, RNA pull-down results suggested that hnRNPA2B1 could not interact with miR-30b-5p (Figure S5I), as was also indicated by the absence of miR-30b-5p in the hnRNPA2B1 IP product (data not shown). These results suggested that miR-30b-3p could be more effectively packaged into H-EVs through hnRNPA2B1.

Next, we investigated how hypoxia induced EV-miR-30b-3p through hnRNPA2B1. Although hypoxia elevated cytoplasmic hnRNPA2B1 levels, it could not induce its nucleocytoplasmic translocation (Figure S5J-K). As shown in Figure S5L, hypoxia treatment could induce hnRNPA2B1 expression in GSCs (Figure S5L), consistent with the results that hnRNPA2B1 overexpression could increase EV-miR-30b-3p (Figure 4D).

Together, our results suggested that increased miR-30b-3p in H-EV was due to two factors. First, hypoxia-induced HIF1a and STAT3 activation in GSCs could promote MIR30B transcription, thereby increasing mature miR-30b-3p and 5p in GSCs. And second, hypoxia-induced hnRNPA2B1 could interact with miR-30b-3p and not miR-30b-5p, contributing to effective packaging of miR-30b-3p into EVs.

### MiR-30b-3p directly targets ras homolog family member B (RHOB) in GBM cells

**Figure 5:**
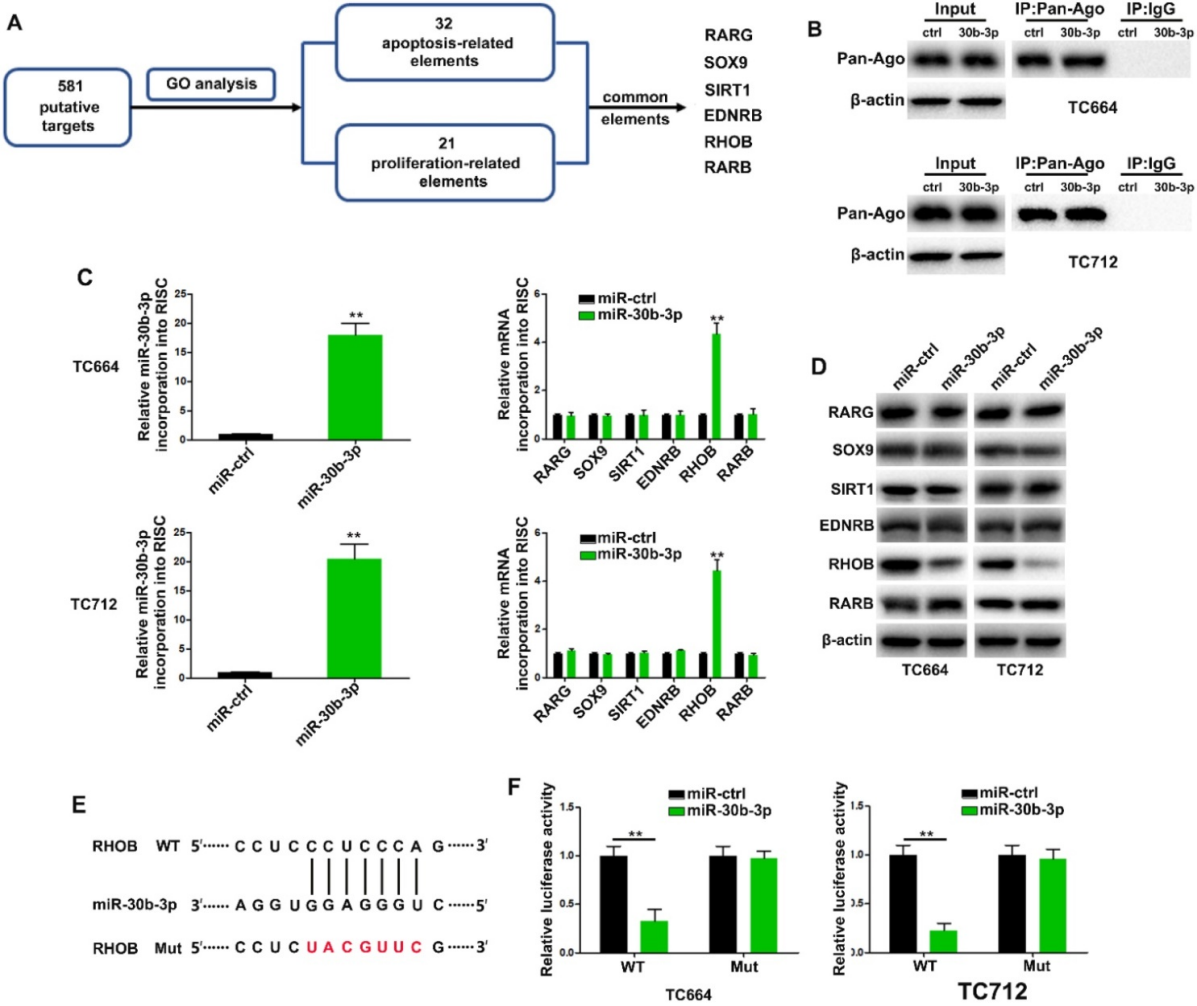
** MiR-30b-3p directly targets RHOB in GBM cells.** (A) Diagram showing the predicted candidate target genes of miR-30b-3p by Gene Ontology analysis. (B) Immunoprecipitation of the Ago2/RISC (RNA-induced silencing complex) using the Pan-Ago2 antibody in TC664 and TC712 cells overexpressing miR-ctrl or miR-30b-3p. IgG was used as a negative control and β-actin was used as an internal control. Three replicates per group, three independent experiments per group. (C) Left: qRT-PCR analysis of miR-30b-3p incorporated into RISC in TC664 and TC712 cells overexpressing miR-30b-3p compared to the levels in the control. U6 RNA was used as an internal control; Right: qRT-PCR of RHOB incorporated into RISC in TC664 and TC712 cells overexpressing miR-30b-3p. GAPDH RNA was used as an internal control. Three replicates per group, three independent experiments per group. (D) Western blot analysis of 6 putative targets of miR-30b-3p. Three replicates per group, three independent experiments per group. (E) Predicted miR-30b-3p target sequence in RHOB-3′ UTRs. Target sequences of RHOB-3′ UTRs were mutated. (F) Luciferase assay of TC664 and TC712 cells transfected with RHOB-3′ UTR-WT or RHOB-3′ UTR-Mut reporter together with miR-ctrl or miR-30b-3p mimic. Six replicates per group, three independent experiments per group, **P < 0.01.

Using miRecords, we found 581 putative targets of miR-30b-3p predicted by at least 5-11 databases. We then performed gene ontology analysis to curate potential genes regulating chemoresistance phenotypes. The results revealed 32 and 21 genes related to apoptosis and proliferation, respectively, among which six were common between the two gene sets (Figure 5A). The RNA-ChIP analysis was used to determine mRNAs selectively abundant in the Ago2/RISC complex following miR-30b-3p overexpression (Figure 5B). RHOB was significantly enriched in RISC from TC664 and TC712 cells overexpressing miR-30b-3p (Figure 5C), while the other five mRNAs showed only slight or no enrichment. Also, RHOB levels were significantly down-regulated after miR-30b-3p overexpression (Figure 5D). The dual-luciferase reporter assay showed that wild-type miR-30b-3p binding sites of the 3′UTR of RHOB led to decreased luciferase activity relative to mutated binding sites (Figure 5E-F). To further analyze the role of miR-30b-3p/RHOB signaling in TMZ resistance, we co-transfected TC664 and TC712 cells with miR-30b-3p mimics and RHOB. Enforced RHOB expression reversed the induced TMZ resistance caused by miR-30b-3p overexpression (Figure S6A-D). Importantly, miR-30b-3p overexpression could increase the IC50 of TCs to TMZ, while RHOB could partially impair this effect (Figure S6E). These results suggested that RHOB was a direct and functional target of miR-30b-3p.

### MiR-30b-3p/RHOB induces chemoresistance *via* bcl-2/bax-mediated apoptosis and CDK2/CDK6-induced cell cycle arrest

**Figure 6:**
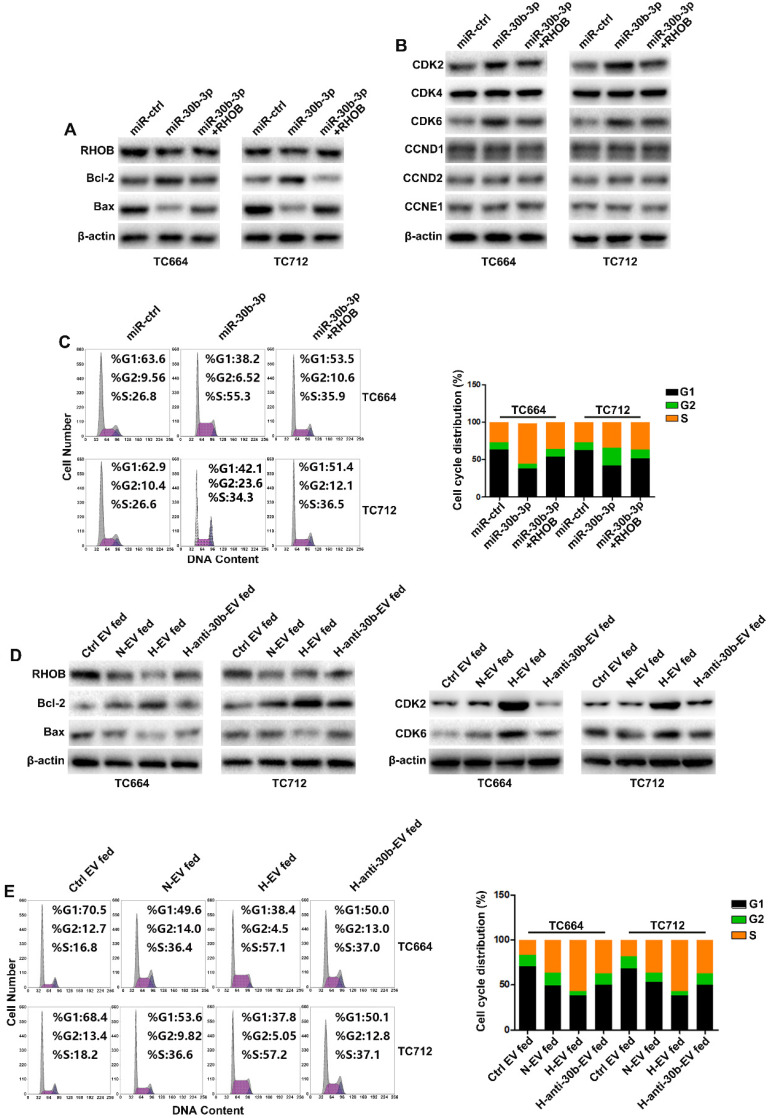
** MiR-30b-3p/RHOB induces chemoresistance via bcl-2/bax-mediated apoptosis and CDK2/CDK6 cell cycle arrest.** (A) Western blot analysis of RHOB, Bcl-2, Bax and β-actin in GBM cells transfected with miR-ctrl, miR-30b-3p, or miR-30b-3p plus RHOB after TMZ treatment (200 µM) for 48 h. Three replicates per group, three independent experiments per group. (B) Western blot analysis of cell cycle regulators (CDK2, CDK4, CDK6, CCND1, CCND2, CCNE1) in GBM cells transfected with miR-ctrl, miR-30b-3p, or miR-30b-3p plus RHOB upon TMZ treatment (200 µM) for 48 h. Three replicates per group, three independent experiments per group. (C) Cell cycle assay of GBM cells transfected with miR-ctrl, miR-30b-3p or miR-30b-3p plus RHOB upon TMZ treatment (200 µM) for 48 h. Six replicates per group, three independent experiments per group. (D) Left: western blot analysis of RHOB, Bcl-2, Bax and β-actin; Right: western blot analysis of CDK2, CDK6, Bax and β-actin in GBM cells pretreated with Ctrl EV, N-EV, H-EV or H-anti-30b-EV upon TMZ treatment (200 µM) for 48 h. Three replicates per group, three independent experiments per group. (E) Cell cycle assay of GBM cells pretreated with Ctrl EV, N-EV, H-EV or H-anti-30b-EV upon TMZ treatment (200 µM) for 48 h. Six replicates per group, three independent experiments per group, **P < 0.01.

TMZ treatment increases apoptosis and cell cycle arrest 35. Gene ontology analysis indicated that RHOB was associated with apoptosis and proliferation (Figure 5A). As shown in Figure 6A, miR-30b-3p overexpression significantly decreased the pro-apoptosis factor bax expression while increasing the anti-apoptosis factor bcl-2 level. Flow cytometry analysis showed that overexpression of miR-30b-3p inhibited the percentage of cells in the G0/G1 phase and increased the percentage of cells in the S and G2/M phases (Figure 6C). We measured the expression of G0/G1 phase regulators to investigate the molecular mechanism involved in the cell cycle block. MiR-30b-3p overexpression significantly increased CDK2 and CDK6 expression, and the forced expression of RHOB reversed miR-30b-3p-induced effects on cell cycle, CDK2, and CDK6 (Figure 6B-C).

We examined the effect of EV-miR-30b-3p on apoptosis and cell cycle following TMZ treatment. EV-miR-30b-3p from hypoxic GSCs significantly suppressed cell apoptotic rate by increasing bcl-2 and decreasing bax, and reduced cell cycle arrest in the G1 phase by upregulating CDK2 and CDK6 (Figure 6D-E). Furthermore, we also analyzed the effect of miR-30b-3p/RHOB on TCs without TMZ treatment. As shown in Supplementary Figures 7A and 7C, miR-30b-3p overexpression significantly promoted cell cycle progression without TMZ treatment. When we measured apoptosis of TCs after overexpression miR-30b-3p, TCs exhibited low apoptotic rate without TMZ treatment, and miR-30b-3p could not induce any change in apoptosis (data not shown). Moreover, EVs from hypoxic GSCs could also promote cell cycle progression in TCs (Figure S7B and S7D) with no change in apoptosis (data not shown). These results indicated that without TMZ treatment, GSCs could promote GBM growth by exosomal transfer of miR-30b-3p. Upon treatment with TMZ, GSCs could maintain tumor growth and confer chemoresistance to TCs by EV-mediated transfer of miR-30b-3p to inhibit apoptosis.

These results collectively suggested that EV-miR-30b-3p suppressed RHOB expression to decrease apoptosis induced by TMZ and decreased cell cycle arrest with or without TMZ treatment.

### Hypoxic EVs enhance tumorigenicity and chemoresistance *in vivo*

**Figure 7:**
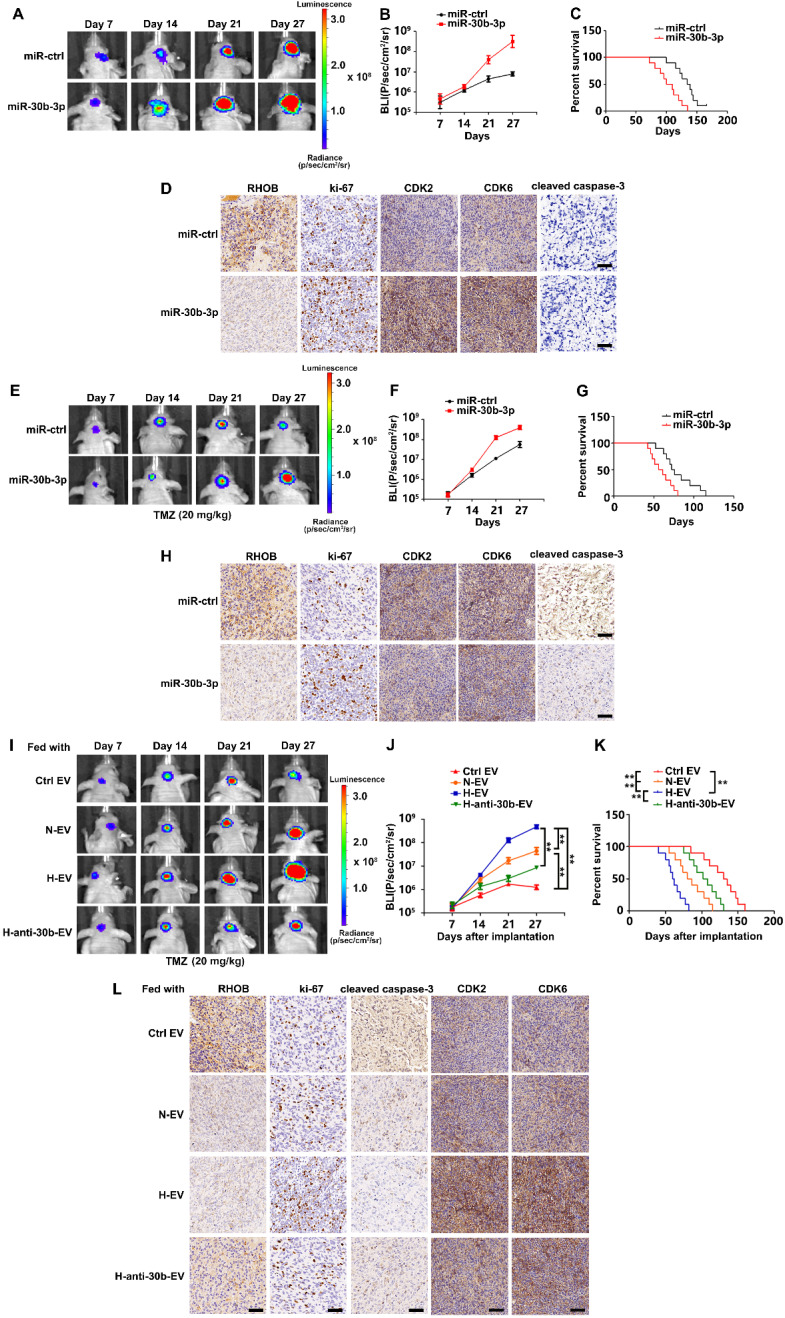
** Hypoxic EVs enhance GSC tumorigenicity and chemoresistance *in vivo.***(A) Pseudocolor bioluminescence images of orthotopic tumors derived from TC664 or miR-30b-3p-overexpressing TC664. n = 10 each group. (B) Bioluminescence was quantified in tumors from four groups. (C) Survival curves of orthotopic tumors derived from TC664 or miR-30b-3p-overexpressing TC664. (D) Immunohistochemical analysis of RHOB, Ki-67, CDK2, CDK6, and cleaved caspase-3 expression in intracranial tumors originating from TC664 or miR-30b-3p-overexpressing TC664. Scale bar, 50 µm. Three replicates per group, three independent experiments per group. (E) Pseudocolor bioluminescence images of orthotopic tumors derived from TC664 or miR-30b-3p overexpressing TC664 with TMZ treatment. n = 10 each group. (F) Bioluminescence was quantified in tumors from four groups with TMZ treatment. (G) Survival curves of orthotopic tumors derived from TC664 or miR-30b-3p-overexpressing TC664 with TMZ treatment. (H) Immunohistochemical analysis of RHOB, Ki-67, CDK2, CDK6, and cleaved caspase-3 expression in intracranial tumors originating from TC664 or miR-30b-3p-overexpressing TC664 with TMZ treatment. Scale bar, 50 µm. Three replicates per group, three independent experiments per group. (I) Pseudocolor bioluminescence images of orthotopic tumors derived from TC664 pretreated with Ctrl EV, N-EV, H-EV, or H-anti-30b-EV upon TMZ treatment (20 mg/kg). n = 10 each group. (J) Bioluminescence was quantified in tumors from four groups. (K) Survival curves of orthotopic tumors derived from TC664 pretreated with Ctrl EV, N-EV, H-EV, or H-anti-30b-EV upon TMZ treatment (20 mg/kg). (L) Immunohistochemical analysis of RHOB, Ki-67, CDK2, CDK6, and cleaved caspase-3 expression in intracranial tumors originating from TC664 pretreated with Ctrl EV, N-EV, H-EV, or H-anti-30b-EV upon TMZ treatment (20 mg/kg). Scale bar, 50 µm. Three replicates per group, three independent experiments per group, **P < 0.01.

We orthotopically injected TC664 cells previously infected with lentivirus expressing luciferase and stably overexpressing miR-30b-3p or miR-NC, into immunocompromised mice. Consistent with the results of *in vitro* experiments, miR-30b-3p overexpression could promote tumor progression by decreasing RHOB and increasing Ki-67, CDK2, and CDK6. Subsequently, we treated mice with TMZ (20 mg/kg/day) or placebo for 5 days per week for three cycles. As is evident from Figures 7E and F, miR-30b-3p overexpression significantly promoted tumor growth. Survival analysis also demonstrated a poor outcome in mice implanted with TC664 cells overexpressing miR-30b-3p (Figure 7G). Xenografts overexpressing miR-30b-3p had significantly decreased RHOB levels, as well as increased levels of Ki-67 and decreased cleaved caspase 3 compared to control xenografts (Figure 7H). These results showed that miR-30b-3p overexpression induced the development of acquired resistance to TMZ *in vivo*.

We assessed the effects of hypoxic EVs *in vivo*. TC712 cells were pre-treated with hypoxic EVs and intracranially implanted into nude mice. Bioluminescence imaging and survival analysis data indicated that GSC EVs significantly contributed to the acquired resistance, tumor growth, and poor survival, and the effect of hypoxic EVs was stronger than normoxic EVs. Besides, down-regulation of miR-30b-3p significantly impaired the effect of hypoxic EVs (Figure 7I-K). These observations were further confirmed by IHC staining data displayed in Figure 7L. Thus, hypoxic GSC EVs induced the development of acquired resistance to TMZ *in vivo*.

### MiR-30b-3p overexpression correlates with poor prognosis and adverse response to TMZ in GBM patients

**Figure 8:**
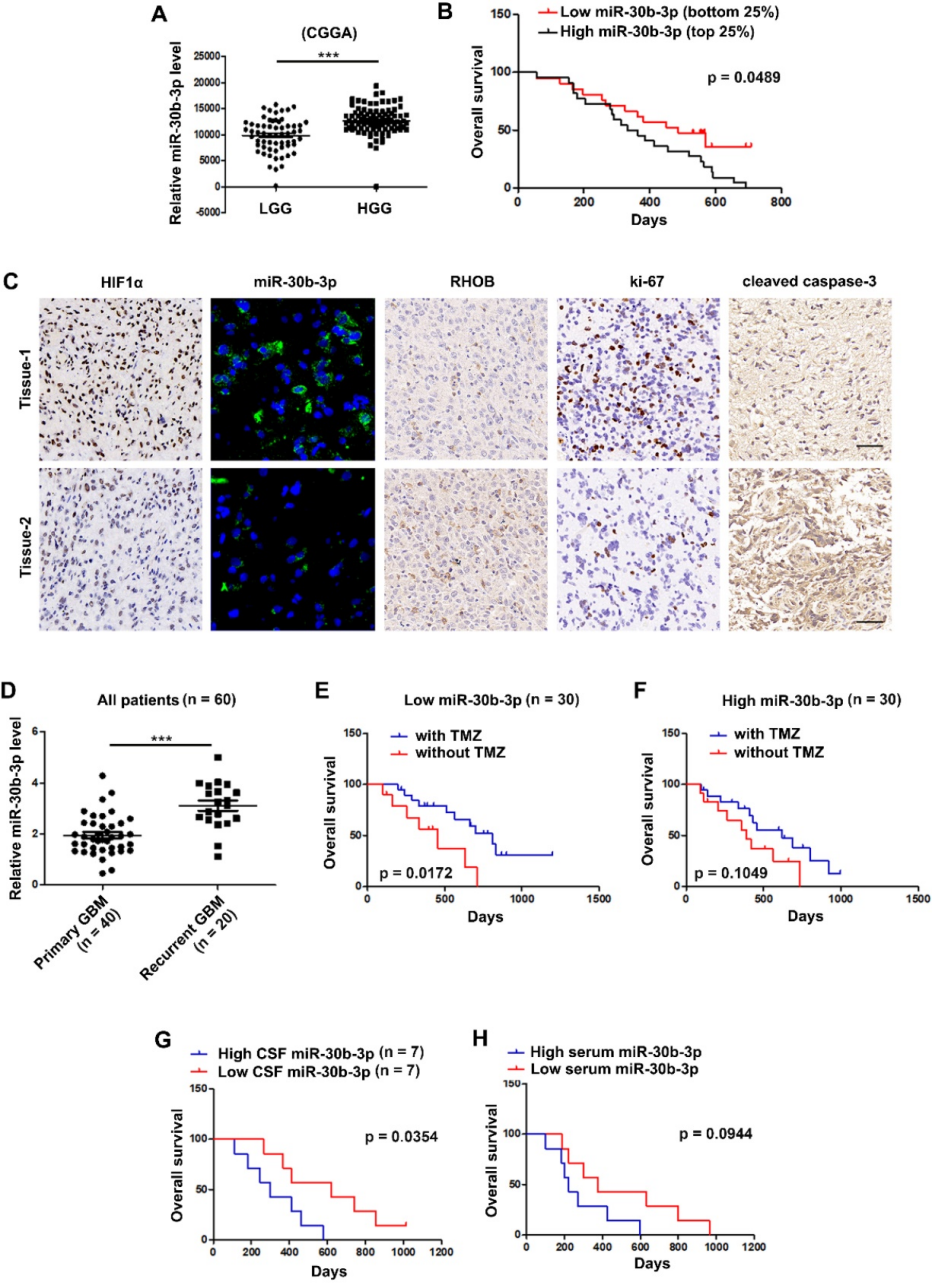
** MiR-30b-3p overexpression correlates with poor prognosis and adverse response to TMZ of GBM patients.** (A) Expression of miR-30b-3p in the Chinese Glioma Genome Atlas (CGGA) cohort of human high-grade and low-grade glioma patients. (B) Overall survival (OS) of GBM patients receiving TMZ treatment with high or low miR-30b-3p expression. (C) HIF1α, miR-30b-3p expression (FISH), RHOB, Ki-67 and cleaved caspase 3 in clinical samples from GBM patients, who had received TMZ-based chemotherapy. Scale bars, 25 µm (FISH) and 50 µm (IHC). Three replicates per sample, three independent experiments per sample. (D) Expression of miR-30b-3p in the clinical samples from primary and recurrent GBM patients. (E) Overall survival (OS) of GBM patients, who received TMZ treatment with low (n = 30) miR-30b-3p expression. (F) Overall survival (OS) of GBM patients, who received TMZ treatment with high (n = 30) miR-30b-3p expression. (G) Overall survival (OS) of GBM patients, who received TMZ treatment with high (n = 7) or low (n = 7) cerebrospinal fluid (CSF) miR-30b-3p expression. (H) Overall survival (OS) of GBM patients who received TMZ treatment with high (n = 7) or low (n = 7) serum miR-30b-3p expression, ***P < 0.001.

We analyzed the expression of miR-30b-3p using the CGGA database and found that miR-30b-3p expression was higher in high grade than in low grade gliomas (Figure 8A). The Kaplan-Meier survival analysis revealed that the bottom 25% of GBM patients with low miR-30b-3p expression showed favorable survival compared with the top 25% of GBM patients with high miR-30b-3p expression (Figure 8B). IHC staining and FISH analysis of GBM clinical samples revealed that tumors with higher miR-30b-3p levels tended to express lower RHOB protein levels and cleaved caspase-3, and higher Ki-67 protein levels (Figure 8C). Furthermore, miR-30b-3p levels in recurrent GBM samples were higher than in primary GBM samples (Figure 8D). Kaplan-Meier analysis of our cohort showed that patients with low miR-30b-3p expression exhibited a prolonged survival after TMZ therapy, while patients with high miR-30b-3p expression had a poor response to TMZ therapy (Figure 8E-F). The results indicated that miR-30b-3p could be a biomarker predicting the response of GBM patients to TMZ treatment. Furthermore, we investigated whether circulating miR-30b-3p could predict TMZ efficacy. As shown in Figure 8G, higher miR-30b-3p levels in the cerebrospinal fluid (CSF) indicated a poor prognosis. However, no significant prognostic difference was observed when patients were evaluated by serum miR-30b-3p (Figure 8H). Thus, the miR-30b-3p level in CSF could serve as an independent predictor for TMZ response in GBM patients.

## Discussion

Intercellular communication within the tumor microenvironment, especially in hypoxia, is an expanding area of central importance to be explored 36. In the hypoxic microenvironment, GSCs, a particular class of cells with infinite proliferation and multi-differentiation potential 30, interact with neurons, astrocytes, vascular cells, and immune cells to support the maintenance of the tumor.

GSCs are believed to play an essential role in glioma heterogeneity, angiogenesis, chemotherapy and radiotherapy resistance, and recurrence 4, 37, 38. However, few studies have focused on GSCs' communication with tumor cells in the context of microenvironment 39-41. Therefore, in-depth studies of the mechanism of GSC-promoted drug resistance are crucial to improve the prognosis of gliomas. In this study, we observed that the culture medium from hypoxic GSCs induced more serious chemoresistance of tumor cells than that from normoxic GSCs. The results suggested a distinct relationship between hypoxic GSCs and tumor cells.

As a well-recognized mediator of cellular communication, EVs have been implicated in various tumor progression processes in multiple cancer subtypes 42, 43. In GBM, EVs were demonstrated to contain miRNA processing machinery and deliver miRNAs that induced transformation and tumor formation in non-tumorigenic mammary cells 10, 26, 44. As a vehicle for message delivery, EVs were reported to transfer diverse materials between different cells. A recent report demonstrated that hypoxic microenvironments stimulated tumor cells to generate miR-21-rich EVs that were delivered to normoxic cells to promote pro-metastatic behavior 45.

Our study found that communication via EVs between GSCs and tumor cells could increase GBM resistance to TMZ. Moreover, hypoxia was a significant stimulator enhancing the effect of GSC-derived EVs. By high-throughput sequencing, we identified miR-30b-3p as an important element in hypoxic EVs compared to normoxic EVs. CHIP and luciferase activity reporter assays showed that miR-30b-3p transcription was dependent upon both HIF1α and STAT3.

Previous studies have shown that the donor cells sort and incorporate specific miRNAs into the EVs, although the underlying mechanisms are far from clear. Four possible routes for miRNA sorting into EVs have been reported: neutral sphingomyelinase-2 (nSMase2)-dependent pathway 46, miRNA motifs and heterogeneous nuclear ribonucleoproteins (hnRNPs)-dependent pathway 34, 47**,** 3′-end of miRNA sequence-dependent pathway 48**,** and miRNA-induced silencing complex (miRISC)-related pathway 49. In this study, we found that miR-30b-3p harbored a potential hnRNPA2B1-binding site. We confirmed the interaction between miR-30b-3p and hnRNPA2B1 by RIP and RNA pull-down assays. Also, silencing hnRNPA2B1 significantly decreased the EV-miR-30b-3p expression level in GSC EVs. The results demonstrated that the interaction with hnRNPA2B1 could increase miR-30b-3p presence in GSC-derived EVs and add a new dimension to the possible sorting mechanisms for miRNA packaging into EVs.

Our earlier studies suggested that EV-miRNAs function by targeting specific genes 24, 27. In this study, we demonstrated that EVs from hypoxic GSCs containing miR-30b-3P significantly down-regulated the tumor-suppressor RHOB in the recipient tumor cells. RHOB belongs to the Rho small GTPase family and functions as a critical regulator of cell-cycle progression and apoptosis 50, 51. RHOB is generally downregulated and functions as a tumor suppressor in various tumor types. However, recent studies provided new perspectives. For example, RHOB was upregulated in breast cancer compared to adjacent normal tissues 52. In GBM, no significant difference in RHOB was found between tumor tissues and normal brain tissues. Meanwhile, RHOB utilize oncogenic shape in GBM cells 50. It was also reported that hypoxia could activate RHOB through glycogen synthase kinase-3 in GBM cells 53. Clearly, a better mechanistic insight of RhoB's role in GBM cells and in GSCs is needed. In this context, our findings demonstrated that EV-miR-30b-3p directly targeted RHOB resulting in decreased apoptosis via increasing bcl-2 and decreasing bax and reduced cell cycle arrest in the G1 phase by increasing CDK2 and CDK6. The results suggested a potential anti-oncogenic role of RHOB in GBM cells.

Recurrence is a serious problem for GBM treatment. Besides regular imaging examination, the biomarkers for tracking tumor evolution through liquid biopsies are an emerging area for monitoring tumor progression and recurrence. Previous study has proved the potential role of EVs as biomarkers for the diagnosis of glioma 54, 55. In this context, CSF possesses unique and intrinsic advantages due to its direct communication with brain tumors. To date, there are very few studies exploring biomarkers in CSF for detection, follow-up, or prognostication of gliomas. CSF-derived EVs contain specific RNA signatures reflecting the underlying molecular genetic status of EGFR vIII mutation in gliomas 56. Alexander et.al demonstrated that miR-15b and miR-21 in CSF could serve as novel biomarkers for glioma detection 57. These studies suggested that CSF could be utilized for diagnosing and monitoring gliomas. In this study, we identified miR-30b-3p, the higher expression of which in CSF indicated poor response of GBM patients to TMZ.

In summary, we found that under hypoxia, HIF-1α and STAT3 transcriptionally induced miR-30b-3p expression in GSCs. HnRNPA2B1 specifically interacted with miR-30b-3p, resulting in its incorporation into EVs. In recipient GBM cells, EV-miR-30b-3p directly targeted RHOB and promoted TMZ resistance. Higher levels of miR-30b-3p in tumor tissues and CSF of GBM patients indicated poor response to TMZ. These results provide a potential biomarker for predicting the response of GBM patients to chemotherapy. However, in-depth analyses of other EV components as well as more clinical data are needed to verify our results.

## Supplementary Material

Supplementary figures and tables.

## Abbreviations

EVs: extracellular vesicles
GBM: glioblastoma
GSCs: glioma stem-like cells
TMZ: temozolomide
miR-30b-3p: microRNA-30b-3p
HIF: hypoxia inducible factor
STAT3: Signal transducer and activator of transcription 3
RHOB: ras homolog family member B
CSF: cerebrospinal fluid
