# Supplementary material for: Extracellular vesicles derived from hypoxic glioma stem-like cells confer temozolomide resistance on glioblastoma by delivering miR-30b-3p
Source: Theranostics. 2021 Jan 1;11(4):1763–79. doi: 10.7150/thno.47057 (PMC7778586; doi:10.7150/thno.47057)
Supplement: Supplementary file 1 — Supplementary figures and tables. [file thnov11p1763s1.pdf]

Supplementary figure legends

Figure S1

(a) Flow cytometry results of CD133 positive cells from primary cells.

(b) Representative immunofluorescence staining images of CD133 (red) and Nestin (green) in GSC664 and GSC712 neurospheres. Scale bar, 100 μm.

(c) Western blot analysis of Sox2, Olig2 and GFAP in GSC and TC pairs. Three replicates per group, three independent experiments per group.

(d) qRT-PCR analysis of Nestin, Sox2 and Olig2 in GSC and TC pairs. Six replicates per group, three independent experiments per group.

(e) In vitro limiting dilution assay of GSC664 and GSC712.

(f) Pseudocolor bioluminescence images of orthotopic tumors derived from TCs and GSCs.

(g) The weight of tumors from TCs and GSCs were analyzed.

(h) Survival curves of orthotopic tumors derived from TCs and GSCs.

(i) Immunohistochemical analysis of GFAP and CD31 in tumors derived from TCs and GSCs.

(j) TMZ IC50 of GSC664, GSC712 and TC664, TC712 cells determined by constructing a dose-response curve.

Figure S2

(a) Colony formation assays of TC664 and TC712 cells treated with different conditioned media for 48 h. (conditioned media/ recipient cells). Six replicates per group, three independent experiments per group.

(b) The apoptotic rates of TC664 and TC712 cells treated with different conditioned media for 48 h were measured by flow cytometry. (conditioned media/ recipient cells). Six replicates per group, three independent experiments per group.

(c) The apoptotic rates of TC664 and TC712 cells treated with different conditioned media for 48 h were measured by TUNEL analysis. (conditioned media/ recipient cells). Six replicates per group, three independent experiments per group.

(d) Western blot analysis of caspase-3 (full length and cleaved) in TC664 and TC712 cells treated with different conditioned media for 48 h. (conditioned media/ recipient cells). Three replicates per group, three independent experiments per group.

(e) The proliferation of TC664 and TC712 cells treated with different conditioned

media for 48 h were measured by MTT analysis. (conditioned media/ recipient cells). Six replicates per group, three independent experiments per group.

(f) The proliferation of TC664 and TC712 cells treated with different EVs were measured by MTT analysis. Six replicates per group, three independent experiments per group.

**$P < 0.01$

Figure S3

(a) qRT-PCR analysis of miR-30b-3p in 5 paired TCs and GSCs under normoxia and hypoxia. Six replicates per sample, three independent experiments per sample.

(b) qRT-PCR analysis of miR-30b-3p in EVs from GSC574, GSC679 and GSC728 cells under normoxia and hypoxia. Six replicates per sample, three independent experiments per sample.

(c) qRT-PCR analysis of miR-30b-3p in EVs and corresponding cells (664), which were treated with anti-miR-30b-3p or negative control, cultured under normoxia and hypoxia. Six replicates per group, three independent experiments per group.

(d) qRT-PCR analysis of miR-30b-3p in EVs and corresponding cells (712), which were treated with anti-miR-30b-3p or negative control, cultured under normoxia and hypoxia. Six replicates per group, three independent experiments per group.

**$P < 0.01$

Figure S4

(a) Western blot analysis of HIF1α, HIF2α, pSTAT3 and STAT3 in GSC664 and GSC712 cells cultured under hypoxia (1% O_2) for indicated amount of time. Three replicates per group, three independent experiments per group.

(b) Western blot analysis of HIF1α, HIF2α, pSTAT3 and STAT3 in GSC664 and GSC712 cells treated with shctrl or shHIF1α or shSTAT3. Three replicates per group, three independent experiments per group.

(c) Relative expression of pri-miR-30b (left) and pre-miR-30b-3p in GSCs were measured. Three replicates per group, three independent experiments per group.

(d) Cartoon of MIR30B promoter with 3 putative hypoxia response elements (HREs) and 3 STAT3 binding sites.

(e) Chromatin immunoprecipitation products for putative HIF1α and STAT3 binding sites were identified by agarose gel electrophoresis.

(f) Schematic representation of the MIR30B promoter reporters.

(g) Western blot analysis of HIF1a, pSTAT3 and STAT3 in GSC664 and GSC712 cells transfected with vector or HIF1a or STAT3. Three replicates per group, three independent experiments per group.

Figure S5

(a) FISH analysis of miR-30b-3p and miR-30b-5p in GSC664. Scale bar, 50 μM.
(b) Gel electrophoresis of qPCR products of miR-30b-3p and miR-30b-5p in subcellular fractions of GSC664 and GSC712 cells. U6 and β-actin acted as nucleus and cytoplasm marker respectively.
(c) 11 potential mediators were detected in pulldown results of miR-30b-3p.
(d) Immunofluorescence analysis of hnRNPA2B1 in GSC664 and GSC712 cells. Scale bar, 50 μM.
(e) Western blot analysis of hnRNPA2B1 in subcellular fractions of GSC664 and GSC712 cells. GAPDH and Histone H3 acted as cytoplasm and nucleus marker respectively.
(f) Western blot analysis of hnRNPA2B1 in N-EV, H-EV and GSCs.
(g) qRT-PCR analysis of miR-30b-3p in normoxic and hypoxic EVs from GSC664 and GSC712 cells transfected with si-Ctrl or si-hnRNPA2B1. Six replicates per group, three independent experiments per group.
(h) qRT-PCR analysis of miR-30b-3p in normoxic and hypoxic EVs from GSC664 and GSC712 cells transfected with vector or hnRNPA2B1. Six replicates per group, three independent experiments per group.
(i) Western blot analysis of hnRNPA2B1 in pulldown production of miR-30b-5p.
(j) Western blot analysis of hnRNPA2B1 in subcellular fractions of hypoxic GSC664 and GSC712 cells. GAPDH and Histone H3 acted as cytoplasm and nucleus marker respectively.
(k) Relative expression of hnRNPA2B1 in subcellular fractions of normoxic and hypoxic GSC664 and GSC712 cells were analyzed.
(l) Western blot analysis of hnRNPA2B1 in normoxic and hypoxic GSCs.
(m) Gel electrophoresis of biotin-labeled wild-type (WT) and mutant (Mut) miR-30b-3p.

Figure S6

(a) Colony formation assays of TC664 and TC712 cells transfected with miR-ctrl or

miR-30b-3p or miR-30b-3p plus RHOB. Six replicates per group, three independent experiments per group.

(b) The apoptotic rates of TC664 and TC712 cells transfected with miR-ctrl, miR-30b-3p or miR-30b-3p plus RHOB were measured by flow cytometry. Six replicates per group, three independent experiments per group.

(c) The apoptotic rates of TC664 and TC712 cells transfected with miR-ctrl, miR-30b-3p or miR-30b-3p plus RHOB were measured by TUNEL analysis. Six replicates per group, three independent experiments per group.

(d) Western blot analysis of caspase-3 (full length and cleaved) in TC664 and TC712 cells transfected with miR-ctrl, miR-30b-3p or miR-30b-3p plus RHOB. Three replicates per group, three independent experiments per group.

(e) TMZ IC50 of TC664 and TC712 cells transfected with miR-30b-3p or co-transfected with miR-30b-3p and RHOB were determined by constructing a dose-response curve.

**$P < 0.01$

Figure S7

(a) Western blot analysis of RHOB, Bcl-2, Bax, CDK2, CDK6 and β-actin in GBM cells transfecting with miR-ctrl, miR-30b-3p or miR-30b-3p plus RHOB without TMZ treatment. Three replicates per group, three independent experiments per group.

(b) Western blot analysis of RHOB, Bcl-2, Bax, CDK2, CDK6 and β-actin in GBM cells pretreated with Ctrl EV, N-EV, H-EV or H-anti-30b-EV without TMZ treatment. Three replicates per group, three independent experiments per group.

(c) Cell cycle assay of GBM cells after transfected with miR-ctrl, miR-30b-3p or miR-30b-3p plus RHOB without TMZ treatment. Six replicates per group, three independent experiments per group.

(d) Cell cycle assay of GBM cells pretreated with Ctrl EV, N-EV, H-EV or H-anti-30b-EV without TMZ treatment. Six replicates per group, three independent experiments per group.

(e) **$P < 0.01$

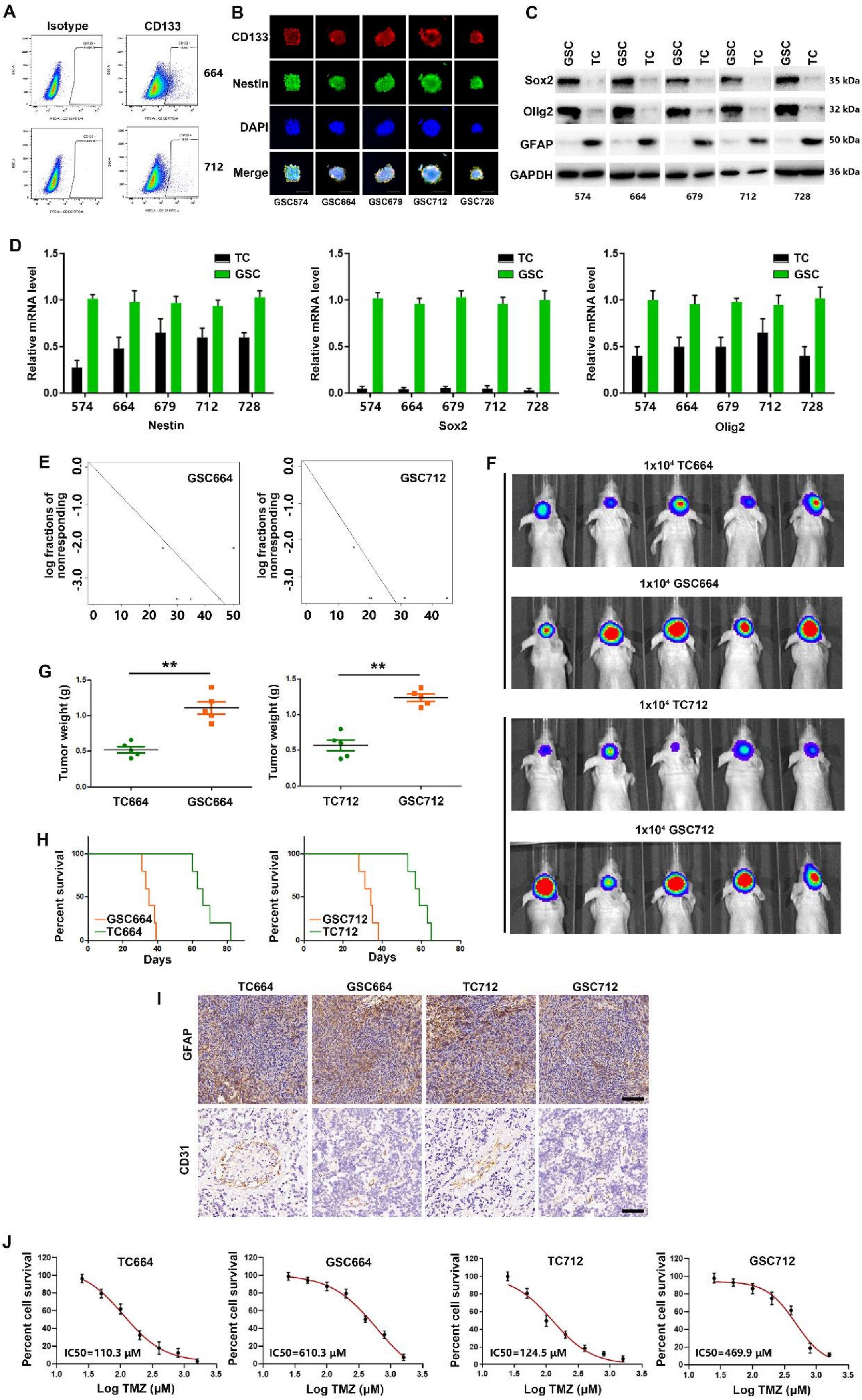
A
Isotype
CD133
664
712
B
CD133
Nestin
DAPI
Merge
GSC574
GSC664
GSC679
GSC712
GSC728
C
GSC
TC
Sox2
Olig2
GFAP
GAPDH
35 kDa
32 kDa
50 kDa
36 kDa
574
664
679
712
728
D
TC
GSC
Relative mRNA level
Nestin
Sox2
Olig2
E
GSC664
GSC712
log fractions of nonresponding
F
1x10⁴ TC664
1x10⁴ GSC664
1x10⁴ TC712
1x10⁴ GSC712
G
Tumor weight (g)
TC664
GSC664
TC712
GSC712
H
Percent survival
Days
GSC664
TC664
GSC712
TC712
I
TC664
GSC664
TC712
GSC712
GFAP
CD31
J
TC664
GSC664
TC712
GSC712
Percent cell survival
IC50=110.3 μM
IC50=610.3 μM
IC50=124.5 μM
IC50=469.9 μM
Log TMZ (μM)

Figure S1

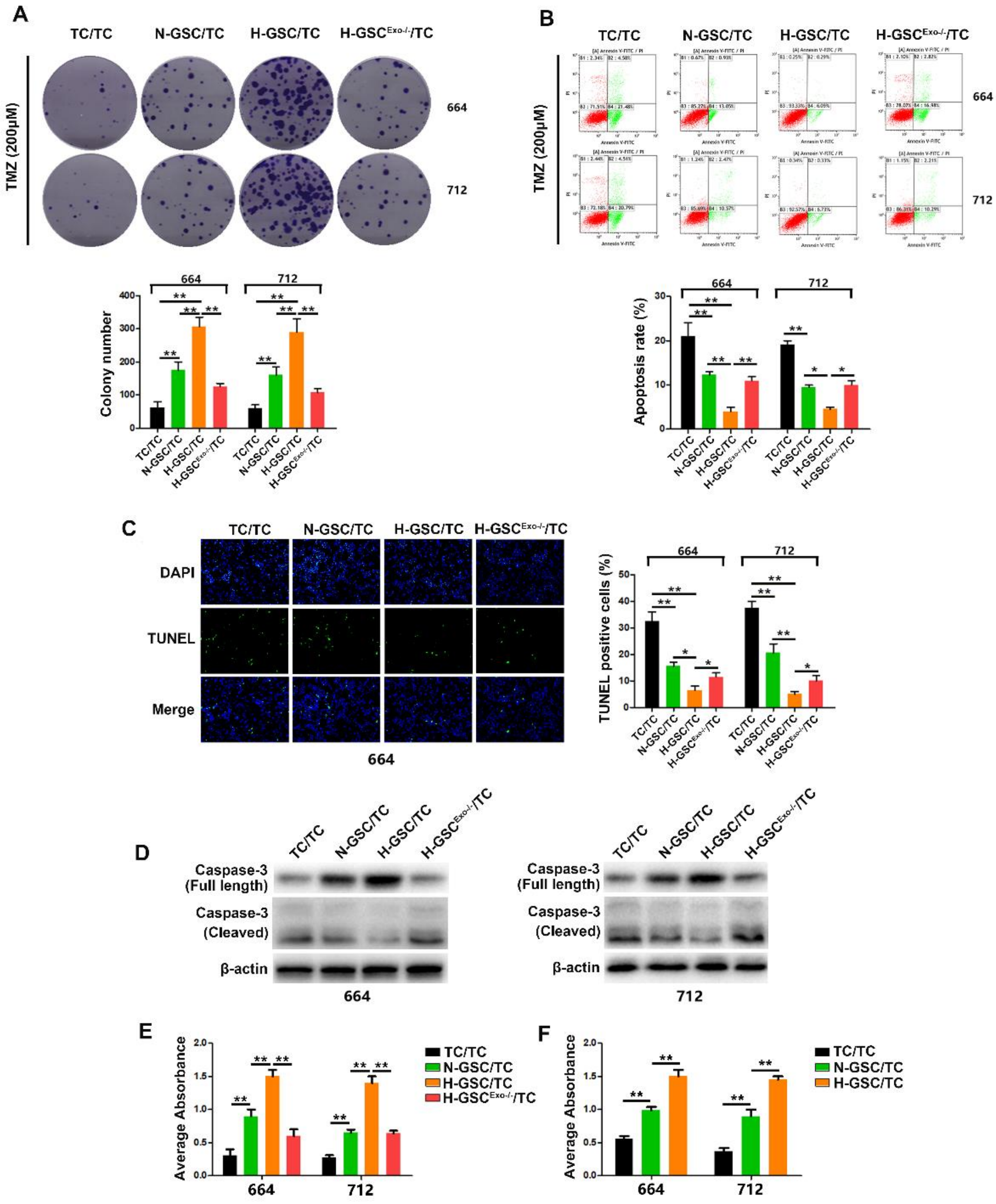
A
TC/TC
N-GSC/TC
H-GSC/TC
H-GSC$^{Exo-/-}$/TC
TMZ (200μM)
664
712
Colony number
B
Apoptosis rate (%)
C
DAPI
TUNEL
Merge
TUNEL positive cells (%)
D
Caspase-3 (Full length)
Caspase-3 (Cleaved)
β-actin
E
Average Absorbance
F

Figure S2

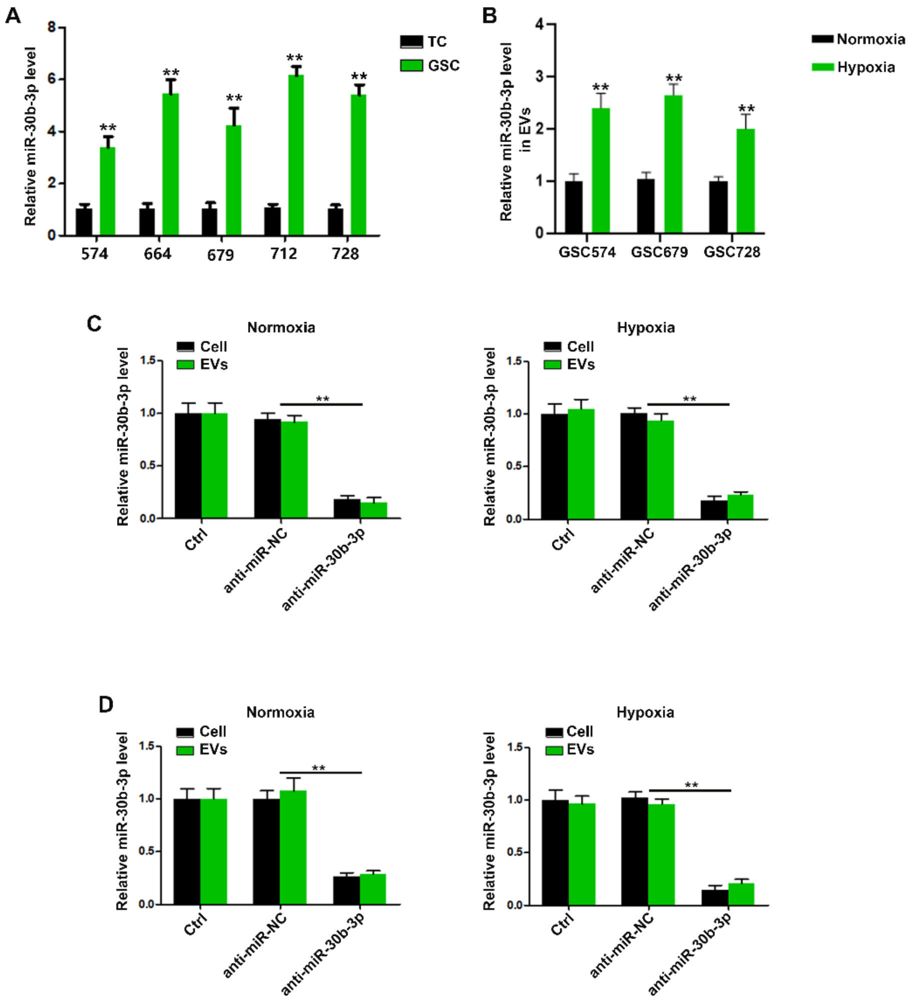
A
Relative miR-30b-3p level
TC
GSC
574
664
679
712
728
B
Relative miR-30b-3p level in EVs
Normoxia
Hypoxia
GSC574
GSC679
GSC728
C
Normoxia
Hypoxia
Cell
EVs
Relative miR-30b-3p level
Ctrl
anti-miR-NC
anti-miR-30b-3p
D
Normoxia
Hypoxia
Cell
EVs
Relative miR-30b-3p level
Ctrl
anti-miR-NC
anti-miR-30b-3p

Figure S3

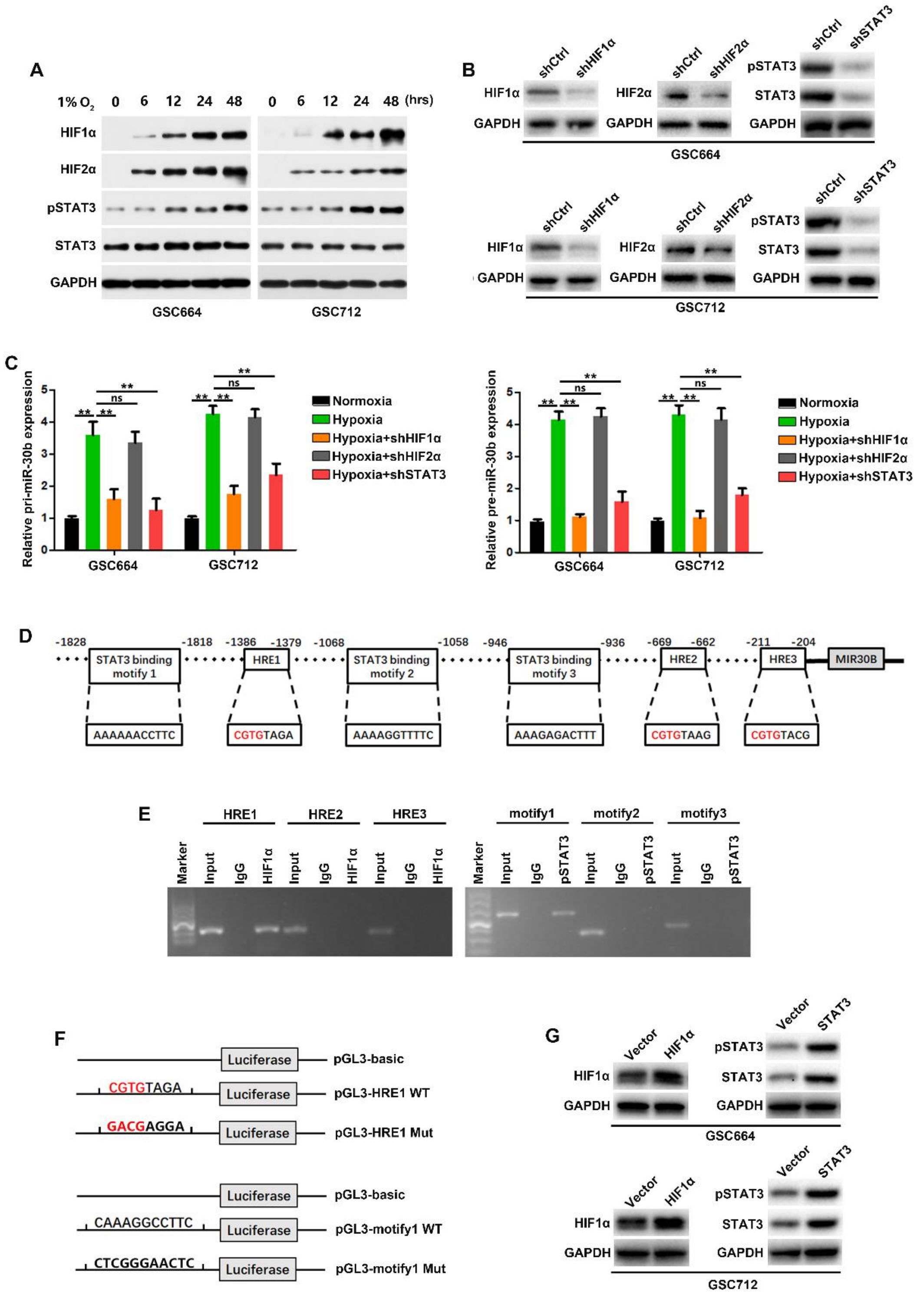
A
1% O2 0 6 12 24 48 0 6 12 24 48 (hrs)
HIF1α
HIF2α
pSTAT3
STAT3
GAPDH
GSC664
GSC712
B
shCtrl shHIF1α
shCtrl shHIF2α
shCtrl shSTAT3
HIF1α
HIF2α
pSTAT3
STAT3
GAPDH
GSC664
GSC712
C
Relative pri-miR-30b expression
Relative pre-miR-30b expression
Normoxia
Hypoxia
Hypoxia+shHIF1α
Hypoxia+shHIF2α
Hypoxia+shSTAT3
GSC664
GSC712
ns
D
-1828 -1818 -1386 -1379 -1068 -1058 -946 -936 -669 -662 -211 -204
STAT3 binding motify 1
HRE1
STAT3 binding motify 2
STAT3 binding motify 3
HRE2
HRE3
MIR30B
AAAAAACCTTC
CGTGTAGA
AAAAGGTTTTC
AAAGAGACTTT
CGTGTAAG
CGTGTACG
E
HRE1 HRE2 HRE3
motify1 motify2 motify3
Marker Input IgG HIF1α
Marker Input IgG pSTAT3
F
Luciferase
pGL3-basic
CGTGTAGA
pGL3-HRE1 WT
GACGAGGA
pGL3-HRE1 Mut
CAAAGGCCTTC
pGL3-motify1 WT
CTCGGGAACTC
pGL3-motify1 Mut
G
Vector HIF1α
Vector STAT3
HIF1α
pSTAT3
STAT3
GAPDH
GSC664
GSC712

Figure S4

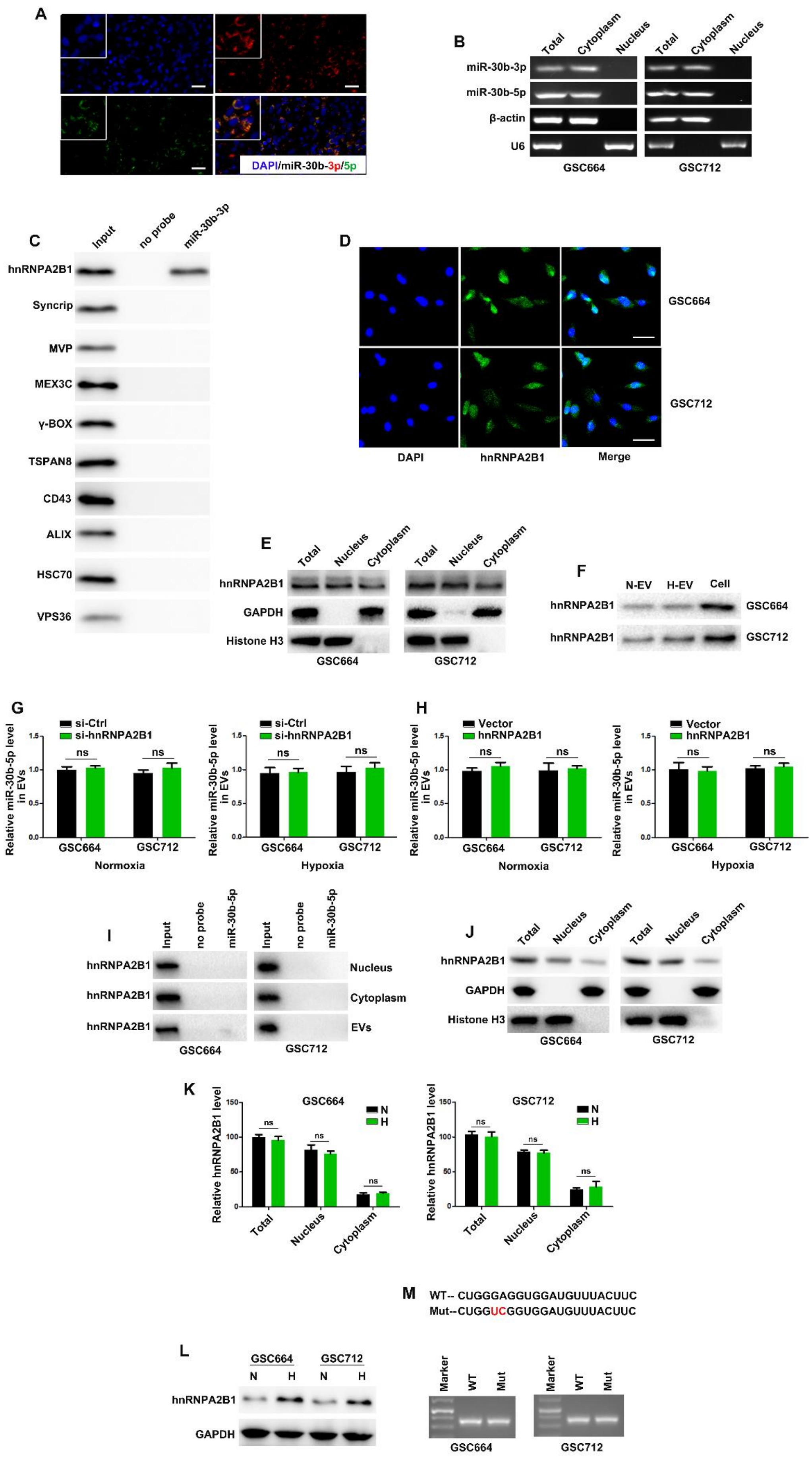
A
DAPI/miR-30b-3p/5p
B
Total
Cytoplasm
Nucleus
miR-30b-3p
miR-30b-5p
β-actin
U6
GSC664
GSC712
C
Input
no probe
miR-30b-3p
hnRNPA2B1
Syncrip
MVP
MEX3C
γ-BOX
TSPAN8
CD43
ALIX
HSC70
VPS36
D
GSC664
GSC712
DAPI
hnRNPA2B1
Merge
E
Total
Nucleus
Cytoplasm
hnRNPA2B1
GAPDH
Histone H3
GSC664
GSC712
F
N-EV
H-EV
Cell
hnRNPA2B1
GSC664
GSC712
G
si-Ctrl
si-hnRNPA2B1
Relative miR-30b-5p level in EVs
ns
GSC664
GSC712
Normoxia
Hypoxia
H
Vector
hnRNPA2B1
I
Input
no probe
miR-30b-5p
Nucleus
Cytoplasm
EVs
J
K
Relative hnRNPA2B1 level
N
H
L
M
WT-- CUGGGAGGUGGAUGUUUACUUC
Mut--CUGGUCGGUGGAUGUUUACUUC
Marker
WT
Mut

Figure S5

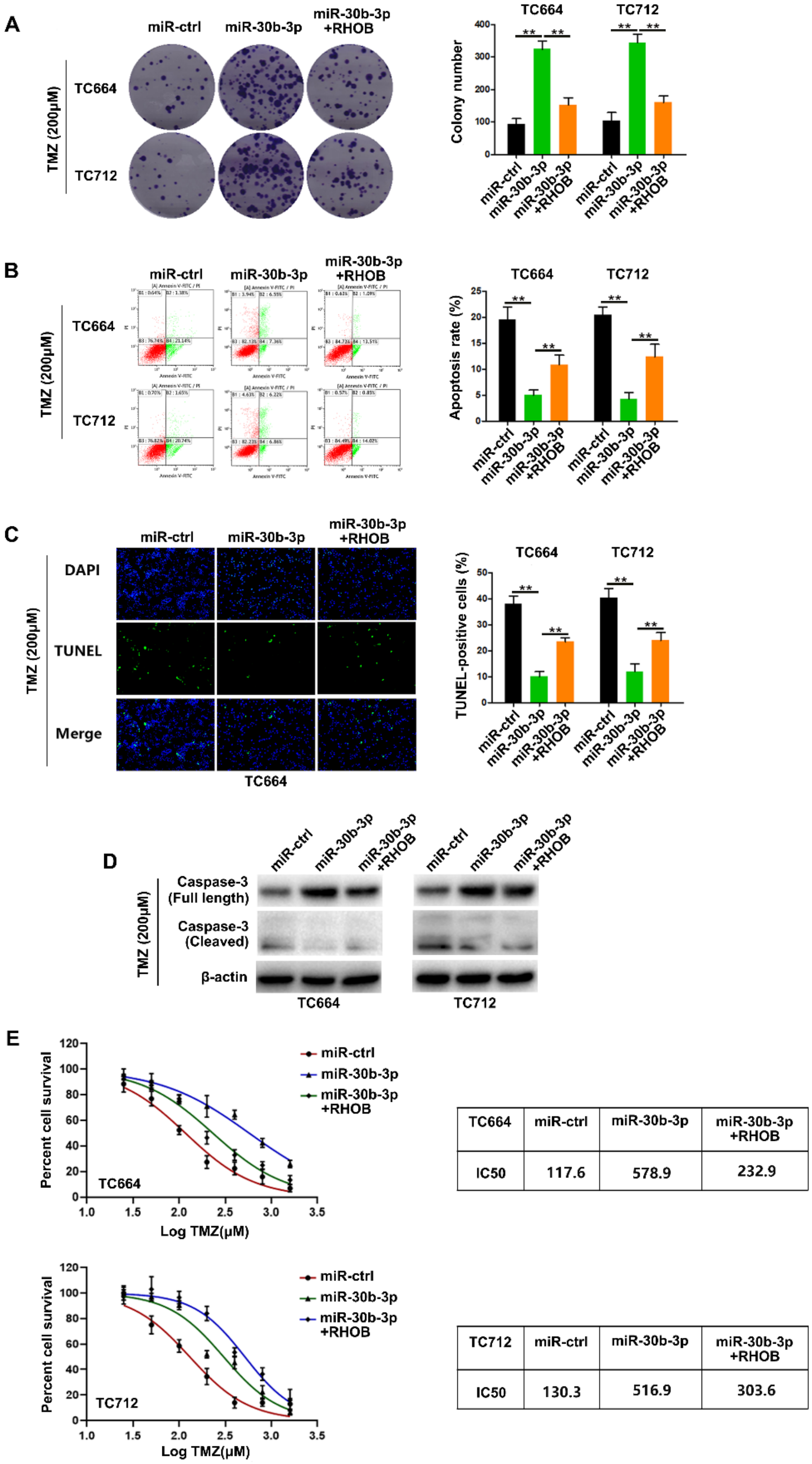

Figure S6

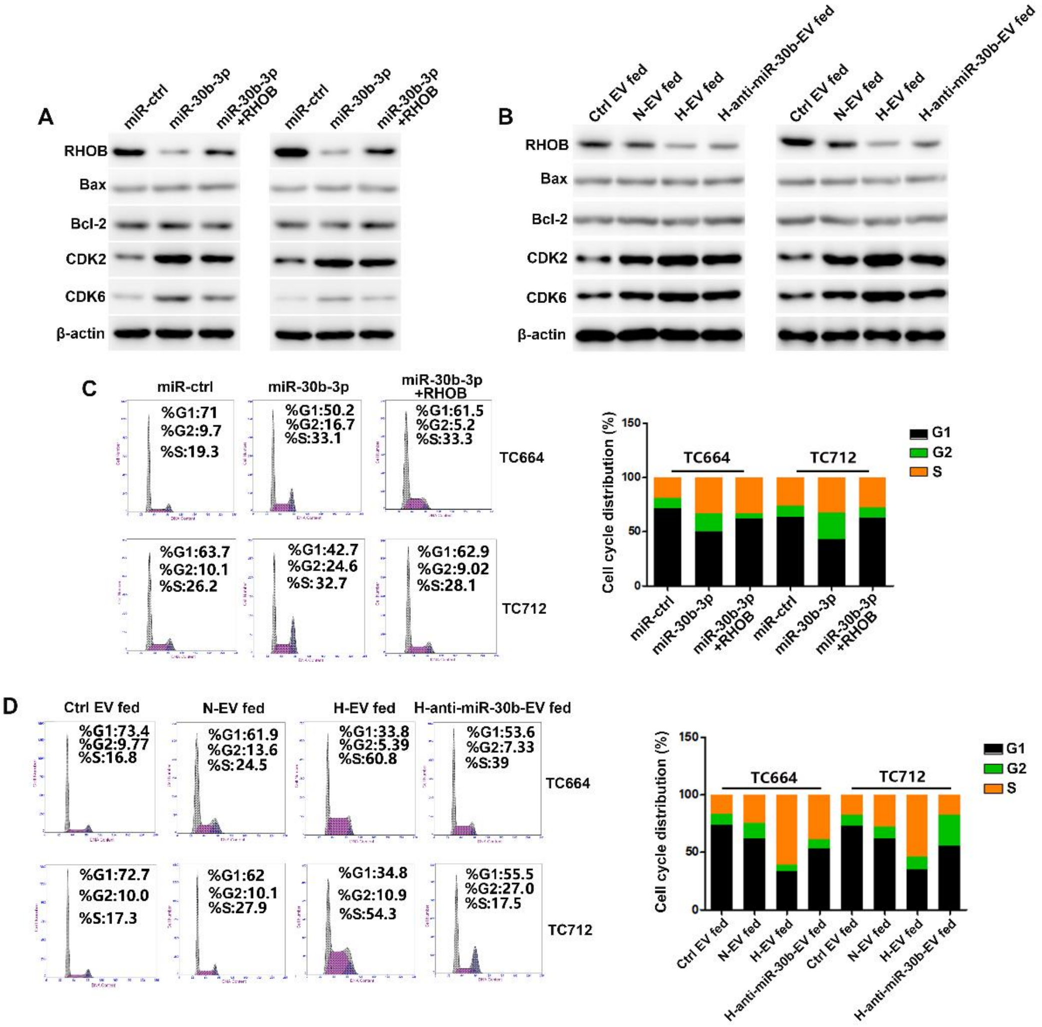
A
miR-ctrl
miR-30b-3p
miR-30b-3p +RHOB
RHOB
Bax
Bcl-2
CDK2
CDK6
β-actin
B
Ctrl EV fed
N-EV fed
H-EV fed
H-anti-miR-30b-EV fed
C
%G1:71
%G2:9.7
%S:19.3
%G1:50.2
%G2:16.7
%S:33.1
%G1:61.5
%G2:5.2
%S:33.3
TC664
%G1:63.7
%G2:10.1
%S:26.2
%G1:42.7
%G2:24.6
%S:32.7
%G1:62.9
%G2:9.02
%S:28.1
TC712
Cell cycle distribution (%)
G1
G2
S
D
%G1:73.4
%G2:9.77
%S:16.8
%G1:61.9
%G2:13.6
%S:24.5
%G1:33.8
%G2:5.39
%S:60.8
%G1:53.6
%G2:7.33
%S:39
%G1:72.7
%G2:10.0
%S:17.3
%G1:62
%G2:10.1
%S:27.9
%G1:34.8
%G2:10.9
%S:54.3
%G1:55.5
%G2:27.0
%S:17.5

Figure S7

Table S1. Clinicopathological features of 60 GBM patients and treatment characteristic

Characteristic	Value
**Total samples (n)**	60
**Sex (n)**	
Male	25
Female	35
**Medium age, years (range)**	54 (38-67)
**Tumor location**	
Frontal	32
Non-frontal	28
**Medium KPS (range)**	78 (65-90)
**MGMT promotor status**	
Methylated	40
Unmethylated	20
**IDH1/2 genotype**	
Mutation	23
Wild-type	37
**EBRT dose (Gy)**	60
**TMZ dose**	75 mg/m^2/d after first surgery

Abbreviations: KPS (Karnofsky performance status); MGMT (O-6-methylguanine-DNA-methyltransferase); IDH1/2 (isocitrate dehydrogenase 1 and 2); TMZ (temozolomide).

Table S2. Status of MGMT in Tumor cells and GSCs

	GSC574 and TC574	GSC664 and TC664	GSC679 and TC679	GSC712 and TC712	GSC728 and TC728
MGMT Methylation	M	UM	M	M	M

M: Methylation; UM: Unmethylation

Table S3. Primers sequence used in this study.

Primers used for quantitative RT-PCR

**Name**	**Forward-primer**	**Reverse-primer**
miR-30b-3p	TGAAAGAGAGAACGATAAATGTT	ACTTCTGAATCAAAATATTGGTA
miR-30b-5p	CCAGCAACTGTAAACATCCTACAC	TATGGTTTTGACGACTGTGTGAT
RHOB	TCTGACCACACTTGTACGCT	GGGGCAGGTGTATTCTCTGT
U6	CCACTGTGGCTTCTGGCATA	ATGAGAGCTTTGGTTCCCCG
β-actin	GTCATTCCAAATATGAGATGCGT	GCATTACATAATTTACACGAAAGCA

Primers used for ChIP-qPCR

**Name**	**Forward-primer**	**Reverse-primer**
HIF1α binding site1	CATCTGCACACAACTTTCCGC	TCTGCACAAACAGACGCCG
HIF1α binding site2	CTGTCTCGGCTGTTATCGCTAC	GCGATACTTGCCACTTAGTA
HIF1α binding site3	CTTCCAGCGGCTGCCTTATCG	GCCGGCTTATCTACCGTCGTA
HIF2α binding site1	CGTTAATGCCCATCGCGTA	TCCATGTCGGATCTAAGCT
HIF2α binding site2	ATCGTAACAGGGACCTAAC	ATGGCAGCAGGTATACGAC
HIF2α binding site3	AGCCTTACATAAGCTTAGC	ATCGTATTCTCCGTAAGTA
pSTAT3 binding site1	TACCATTGCCTGATATCGTA	TAGGCTCTGAAAGGTCTTG
pSTAT3 binding site2	CTAACGCCTATTCGATGCA	GACCTTAAGCCACTGACA
pSTAT3 binding site3	ATCCGTACGATTATTGCGC	TACGCCTACGTTCGTATCA

Table S4. Antibodies used in this study.

Name	Company	Catalog Number	Assay
Sox2	Cell Signaling Technology	#3579	WB
Olig2	Abcam	AB42453	WB
GFAP	Cell Signaling Technology	#80788	WB
CD81	Cell Signaling Technology	#10037	WB
CD63	Abcam	AB217345	WB
Caspase-3	Cell Signaling Technology	#14220	WB
β-actin	Proteintech	Cat.No.60008-1-Ig	WB
HIF1α	Cell Signaling Technology	#36169	WB,CHIP
HIF2α	Cell Signaling Technology	#59973	WB,CHIP
pSTAT3	Cell Signaling Technology	#9145	WB,CHIP
STAT3	Cell Signaling Technology	#12640	WB
GAPDH	Proteintech	Cat.No. 60004-1-Ig	WB
hnRNPA2B1	Abcam	AB31645	RIP,WB
RARG	Sigma-aldrich	#8965	WB
SOX9	Abcam	#82630	WB
SIRT1	Abcam	#2493	WB
EDNRB	Abcam	AB117529	WB
RHOB	Abcam	AB102770	WB
RARB	Abcam	AB53161	WB
Histone H3	Cell Signaling Technology	#4499	WB
